# Glucoregulatory and Cardiometabolic Profiles of Almond vs. Cracker Snacking for 8 Weeks in Young Adults: A Randomized Controlled Trial

**DOI:** 10.3390/nu10080960

**Published:** 2018-07-25

**Authors:** Jaapna Dhillon, Max Thorwald, Natalie De La Cruz, Emily Vu, Syed Asad Asghar, Quintin Kuse, L. Karina Diaz Rios, Rudy M. Ortiz

**Affiliations:** 1 School of Natural Sciences, University of California, Merced, CA 95343, USA; mthorwald@ucmerced.edu (M.T.); ndelacruz2@ucmerced.edu (N.D.L.C.); evu2@ucmerced.edu (E.V.); sasghar@ucmerced.edu (S.A.A.); qkuse@ucmerced.edu (Q.K.); rortiz@ucmerced.edu (R.M.O.); 2 Cooperative Extension Specialist, University of California, Merced, CA 95343, USA; kdiazrios@ucmerced.edu

**Keywords:** C-peptide, HDL cholesterol, lipids, metabolism, nuts, satiety

## Abstract

The transition to nutritional independence makes new college students vulnerable to alterations in eating patterns, which can increase the risk of cardiometabolic disorders. The aim of the study was to examine the potential benefits of almond vs. cracker snacking in improving glucoregulatory and cardiometabolic profiles in new college students. A randomized controlled, parallel-arm, 8-week intervention of 73 college students (BMI: 18–41 kg/m^2^) with no cardiometabolic disorders was conducted. Participants were randomized into either an almond snack group (56.7 g/day; 364 kcal; *n* = 38) or Graham cracker control group (77.5 g/day; 338 kcal/d; *n* = 35). Chronic, static changes were assessed from fasting serum/plasma samples at baseline, and after 4 and 8 weeks. Acute, dynamic effects were assessed during a 2-h oral glucose tolerance test (OGTT) at 8 weeks. Almond snacking resulted in a smaller decline in HDL cholesterol over 8 weeks (13.5% vs. 24.5%, *p* < 0.05), 13% lower 2-h glucose area under the curve (AUC), 34% lower insulin resistance index (IRI) and 82% higher Matsuda index (*p* < 0.05) during the OGTT, despite similar body mass gains over 8 weeks compared with the cracker group. In general, both almond and cracker snacking reduced fasting glucose, and LDL cholesterol. Conclusions: Incorporating a morning snack in the dietary regimen of predominantly breakfast-skipping, first-year college students had some beneficial effects on glucoregulatory and cardiometabolic health. Almond consumption has the potential to benefit postprandial glucoregulation in this cohort. These responses may be influenced by cardiometabolic risk factor status.

## 1. Introduction

The benefits of nut consumption in ameliorating cardiovascular disease [1] and reducing the risk of type 2 diabetes mellitus (T2DM) [2] in adults are well established. A meta-analysis found an inverse association between nut consumption and diabetes prevalence, which became non-significant after adjusting for BMI [1] suggesting that the effects of nut consumption on diabetes prevalence are largely mediated by changes in body mass (adiposity). Almond studies demonstrating improvements in glycemic control in T2DM [3,4], impaired glucose-tolerant (IGT) [5] individuals, and in healthy adults [6], have mostly been performed in middle-aged to older adults (median age >40 years). Despite increasing incidence of metabolic disorders (e.g., obesity, insulin resistance) at younger ages [7,8], no evidence exists on the potential benefits of almond consumption in ameliorating metabolic disorders at earlier life stages (e.g., adolescence, young adulthood). Studying young adults, especially those starting college, is clinically relevant because of the potential risk factors derived from changes in eating behavior occurring during the transition from adolescence to adulthood [9,10].

The transition to dietary independence makes new college students vulnerable to unfavorable alterations in eating patterns [11]. For instance, a relatively high number of college freshman report skipping breakfast (20–43%); by far the most skipped meal in this group [9,12,13,14]. In turn, persistently skipping breakfast has detrimental outcomes on cardiometabolic health [15] and academic performance in adolescents [16]. It is known that most college students snack [12] both, in the morning and/or the afternoon [14]. However, although well documented in adults [1,3,5,17], the effects of almond snacking in young college students who routinely skip breakfast are unknown and could have significant implications for diet-related health outcomes in this group. Dietary practices acquired at this developmentally crucial life stage can persist through adulthood, affecting future health outcomes.

Several studies have found the transition to college life to be associated with a modest increase in body mass (BM) of 1–3 kg (2.2–6.6 lb) [18,19]. Other studies have reported high BM gain and prevalence of metabolic syndrome in young adults aged 18–24 years, suggesting that experiences occurring during these years (e.g., transition to college) may affect health behaviors in favor of increased BM and metabolic disorders [20]. While almond-supplemented diets have been shown to reduce BM or ameliorate gain [21,22], the evidence of such effects in young adults transitioning to college is scarce and an untapped area of research.

The purpose of this study was to evaluate the effects of 8 weeks of almond snacking on glucoregulatory and cardiometabolic profiles compared to a snack of Graham crackers in college first years. We employed a study design that included both chronic, static measurements at 4-week intervals (to capture mid-point changes) and acute, dynamic analyses in response to an oral glucose tolerance test (OGTT) after 8 weeks of intervention. We hypothesized that consumption of almonds for 8 weeks improves lipid profile, insulin sensitivity, glucose tolerance and associated hormone profiles in college first years independently of changes in body mass or adiposity.

## 2. Materials and Methods

This study is registered on ClinicalTrials.gov (registration number: NCT03084003). All procedures involving human subjects were approved by the University of California (UC) Merced Institutional Review Board.

### 2.1. Participants

One hundred and twenty-four college first years from UC Merced underwent screening to determine eligibility for participation in the study. Eighty participants were enrolled to participate in the study. Seven participants withdrew prior to study start. Seventy-three (41 women and 32 men) participants (18–19 years old, BMI: 18–41 kg/m^2^) completed the study. Participants were recruited via public advertisements. Informed consent was obtained from participants who met eligibility criteria prior to commencement of study visits. Inclusion criteria included the following: (a) 18–21 years of age, (b) newly enrolled, 1st-year college students, (c) no nut allergies, (d) willingness to consume almonds or Graham crackers, (e) willingness to maintain consistent diet and activity patterns, (f) not taking medications known to influence metabolism and appetite, and (g) non-smoker over the previous year. Exclusion criteria included diagnosed diabetes or pre-diabetes, uncontrolled hypertension, cardiovascular disease or dyslipidemia requiring drug therapy. Obesity was not an exclusion criterion if participants were not on or did not require medications for cardiometabolic disorders. Participants were categorized by BMI and metabolic syndrome risk factors which were assigned according to the International Diabetes Federation (IDF) consensus definition of metabolic syndrome in adolescents [23]. Metabolic syndrome in this cohort was categorized by the presence of central obesity i.e., waist circumference ≥94 cm (Europid males) and ≥80 cm (Europid females)/ethnicity specific values for other groups and two of the following four factors: triglycerides ≥150 mg/dL, HDL cholesterol <40 mg/dL (males) and <50 mg/dL (females), fasting glucose ≥100 mg/dL, and systolic blood pressure ≥130 mm Hg or diastolic blood pressure ≥85 mm Hg [23].

### 2.2. Study Protocol

The study was an 8-week randomized, controlled, parallel-arm intervention. Participants were assigned into one of two study arms: almond snack group (*n* = 40) or control, Graham cracker snack group (*n* = 40) via simple randomization. A blinded researcher used a sequence of computer-generated random numbers to assign participants to group 1 i.e., cracker group or group 2 i.e., almond group. Participants were enrolled to start the study in 2 groups staggered a week apart. Assignments to these groups was random (and not determined by snack group assignments). Participants in the almond group consumed 56.7 g/day (2 oz; 364 kcal; 14% carbohydrate (8 g fiber), 74% fat, 13% protein, Table S1) of dry-roasted almonds and were asked to avoid consumption of other nuts and seeds. The almonds were dry-roasted at 129.4 °C for about 50 min to enhance palatability. Participants in the cracker group consumed 5 sheets (77.5 g/day) of Graham crackers (338 kcal; 74% carbohydrate (2.5 g fiber), 20% fat, 6% protein, Table S1) and were asked to avoid all nuts, seeds, and nut-containing products during the intervention period. The control snack of Graham crackers was selected based on focus groups that were conducted with a subset of participants to determine habitual snack food and intake patterns.

Weekly energy and nutrient intake data was collected using a validated, automated, and self-administered 24-h Dietary Assessment Tool (ASA24) [24]. Participants met with a registered dietitian to receive training on the ASA24 tool; no dietary counseling was provided to either group. Participants met with researchers every day before 12 pm over the study duration (with the exception of weekends and 5-day spring break) to collect and consume their study snacks. Peer-researchers witnessed and recorded participants’ consumption of assigned snack to confirm compliance. Breakfast consumption for the day and sleep duration the previous night was also recorded. Participant compliance over the weekend and spring break was monitored via text messages. Physical activity was tracked every 4 weeks for 2 days: one weekday and one weekend day, using an advanced version of a previously validated triaxial accelerometer (RT6, Stayhealthy Inc., Monrovia, CA, USA) [25].

### 2.3. Study Outcomes

The primary outcomes were glucoregulatory profiles (glucose and insulin) at the end of the 8-week intervention. Secondary outcomes were lipid profile, body mass, body composition, waist circumference, plasma leptin, serum glucagon, serum adiponectin, resting blood pressure, endothelial function, and 24-h free-living appetite. All outcomes were assessed at baseline, 4 and 8 weeks into the intervention except for the OGTT and endothelial function, which were only assessed at week 8.

The sample size calculations for this analysis were based on change in fasting homeostasis model assessment-insulin resistance (HOMA-IR) over time estimated from the change in fasting glucose and fasting insulin concentrations of the control and almond (morning snack) groups of the Tan et al. [17] study. Thirty-five participants were required per snack group to detect 20% change over time from the control with an alpha of 0.05, standard deviation (SD) of 1 and 80% power. The estimated minimum sample size was increased to 40 per group to account for potential dropouts.

#### 2.3.1. Anthropometric Measures

Body mass (kg) and body composition were measured using a calibrated bio-electrical impedance analysis scale (Model BC-418, Tanita Inc., Arlington Heights, IL, USA) with participants wearing minimal light-weight clothing. Height (m) was measured using a wall-mounted stadiometer. BM and height were used to calculate BMI. Waist circumference was measured using a measuring tape placed at the narrowest part of the torso.

#### 2.3.2. Cardiovascular Measures

Resting blood pressure was assessed using an automated digital blood pressure monitor (Model HEM 780, Omron Corporation, Kyoto, Japan). Participants rested for 5 min prior to blood pressure measurement. Two readings were taken, and the mean was calculated to determine resting blood pressure. Reactive Hyperemia Index (RHI), a measure of endothelial function, and augmentation index (AI), a measure of arterial stiffness, were assessed using an Endopat device (Itamar Medical Ltd., Caesarea, Israel).

#### 2.3.3. Biochemical Analyses

Blood samples were collected following an overnight fast of 8–10 h by venipuncture. Blood was collected in chilled spray-coated silica and a polymer gel (BD Vacutainer^®^ SST, 8.5 mL) tubes for serum separation and chilled spray-coated lithium heparin and gel (BD Vacutainer^®^ K_2_EDTA, 6 mL) tubes for plasma separation. Blood samples were immediately separated by centrifuging at 1300–1500 RCF for 10 min at 4 °C. Serum and plasma samples were divided into aliquots and stored at −80 °C until analysis. Consistent assay protocols were used for biochemical measurements over the study period. Serum samples were analyzed for glucose and lipids using the Olympus AU400 (Olympus, Tokyo, Japan) analyzer, and for serum non-esterified fatty acids (NEFAs) (Wako diagnostics HR Series NEFA-HR(2)), insulin (Millipore Human Insulin ELISA EZHI-14K), C-peptide (Mercodia C-peptide ELISA 10-1136-01), glucagon (Mercodia Glucagon ELISA 10-1271-01), GLP-1 (GLP-1 Total ELISA EZGLP1T-36K) and adiponectin (Millipore Human Adiponectin EZHADP-61K). Plasma samples were analyzed for leptin (Millipore Human Leptin EZHL-80SK). Leptin, GLP-1 and adiponectin were measured to assess biochemical markers of energy balance, appetite and insulin action [26,27,28]. C-peptide during the OGTTs was measured to confirm the secretory capacity of the pancreatic β-cells as C-peptide and insulin are secreted in nearly equi-molar amounts [29]. We anticipated that similar patterns between insulin and C-peptide in response to the OGTT would be indicative of β-cell reactivity and less so of systemic insulin metabolism. Results for NEFAs, adiponectin, leptin and glucagon are presented in supplementary material (Figures S1 and S2).

Insulin resistance, secretion, and sensitivity indices were calculated as follows: HOMA-IR ((fasting glucose × fasting insulin)/405) [30], HOMA-β ((360 × fasting insulin)/(fasting glucose-63)) [30] and quantitative insulin sensitivity check (QUICKI) (1/(log_10_ (fasting glucose) + log_10_ (fasting insulin))) [30].

#### 2.3.4. Oral Glucose Tolerance Test

An OGTT was conducted at the end of the 8-week intervention in a subset of 20 participants randomly selected from each snack group. Prior to consuming a 75 g glucose drink (Azer Scientific, Morgantown, PA, USA, 10-FP-075), a blood sample (time 0, T0) was obtained as previously described. Immediately following this collection, participants rapidly consumed their glucose drink and blood samples were obtained at 15, 30, 60 and 120 min after consumption. Blood samples were processed as previously described to obtain serum.

From the OGTTs, the glucose, NEFAs, and hormone AUCs were calculated (described in statistical analyses) as well as insulin resistance index (IRI), Matsuda index (MI) and disposition index (DI). The IRI and MI are measures of whole-body insulin sensitivity. IRI was calculated as glucose AUC_0–120min_ × insulin AUC_0–120min_ [31,32]. MI was calculated as: 10,000/square root of [fasting glucose × fasting insulin] × [mean glucose x mean insulin during OGTT] [33]. The disposition index, a measure of β-cell function was calculated as MI × (*∆*glucose_0–120_ × *Δ*insulin_0–120_) [34].

#### 2.3.5. 24-h Free-Living Appetite Ratings

Hunger, fullness, desire to eat, and prospective consumption ratings were measured on 100-mm visual analog scales with end anchors of “not at all” to “extremely” [35]. These ratings were assessed hourly during waking hours over a 24-h period. The means of the respective appetite ratings over the 24-h period were considered for analysis.

#### 2.3.6. Acceptance and Palatability Ratings

Participants rated the acceptability of their snack biweekly using a 9-point food action rating scale where 1 = “I would eat this if I were forced to” and 9 = “I would eat this every opportunity I had” [36]. Participants rated the palatability of their snack biweekly using a hedonic general labelled magnitude scale (gLMS) [37] where −100 = “Extremely unpalatable” and 100 = “Extremely palatable”.

### 2.4. Statistical Analyses

We conducted a linear, mixed model analysis with week/OGTT time, snack group, and a week/time-by-snack group interaction as factors for all absolute values of outcomes. Data not meeting normality assumptions were transformed using Johnson’s family of transformations. Transformed variables are marked in tables and figure legends. However, only non-transformed data and means are presented for interpretation of biological significance. Analyses were adjusted for baseline/OGTT T0 when baseline/OGTT T0 values had a significant effect on the model. When significant effects were observed, pairwise comparisons were carried out with Tukey’s honest significant difference (HSD), except for OGTT time outcomes, where contrasts between snack groups at different time points were constructed and adjusted for multiple comparisons.

A secondary analysis on the change in outcomes over 8 weeks, as opposed to absolute values, was also performed (Table S2). OGTT AUCs were calculated using the standard trapezoidal rule. Total AUCs were calculated over the entire 120 min as well as over and between the different time points. AUCs and OGTT indices were analyzed by Kruskal-Wallis test with snack group as a between-subject factor. A separate analysis including sex as between-subject factor for change in outcomes over 8 weeks rather than absolute values and AUCs was also conducted but there were no significant differences in any outcome. The effect of baseline BMI category x snack group on change in outcomes over 8 weeks and OGTT AUCs and effect of baseline fasting total cholesterol category x snack group, and baseline fasting glucose category x snack group on change in outcomes over 8 weeks was evaluated using Kruskal-Wallis tests (Tables S3–S5 and S8). Pairwise comparisons were carried out with Dunn all pairs for joint ranks test. A responder analysis was also conducted to determine if the proportion of responders (i.e., those individuals demonstrating a positive change over time for the specific outcome) is different between groups (Table S6).

Partial least squares discriminant analysis (PLS-DA) was conducted on the intervention and OGTT data to identify discriminatory features between the snack groups and principal component analysis (PCA) was conducted on the correlation matrix obtained from the discriminatory features (identified by PLS-DA) for visualization purposes (Figures S3–S5).

The alpha level was set at 0.05. All data are reported as means and SDs unless otherwise stated. The *p*-values for the figures are presented in supplementary material (Table S7). JMP Pro (version 13, SAS Institute Inc., Cary, NC, USA) was used for all the statistical analyses.

## 3. Results

### 3.1. Participant Characteristics

**Figure 1:**
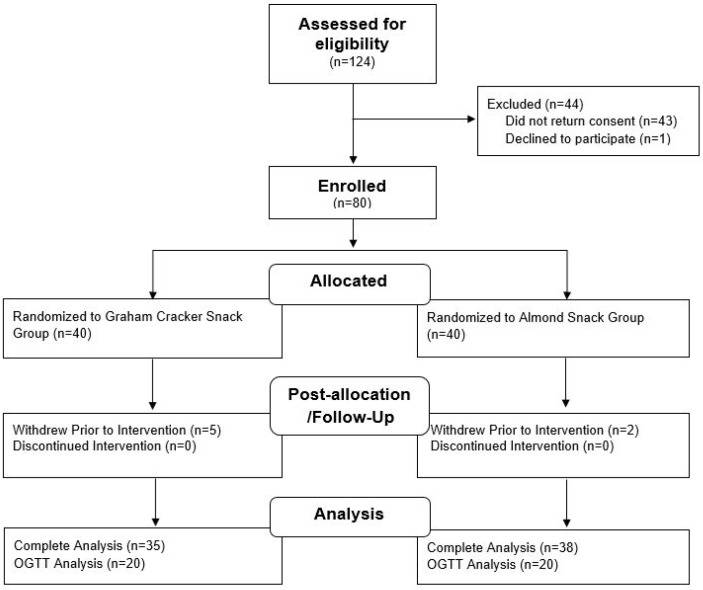
Participant flow throughout the randomized, controlled parallel-arm study. OGTT, oral glucose tolerance test.

Seventy-three participants started and completed the study (Figure 1). The baseline characteristics of all participants are described in Table 1. BMI categories were assigned according to BMI-for-age-percentiles for adolescents [38]. The total cholesterol, LDL and HDL categories are assigned according to healthy cholesterol levels for individuals aged 19 or younger [39]. The proportion of participants with fasting glucose concentration ≥100 mg/dL was higher in the cracker group compared to the almond group (*p* < 0.05, Table 1). In general, study participants were at low to moderate risk of developing cardiometabolic disorders.

### 3.2. Overall Breakfast Consumption and Sleep Habits

Forty-six (63%) participants reported consuming breakfast either rarely, never or between 2–4 times a week prior to the study. During the study, participants reported breakfast consumption (before snack) on an average of 3 days per week (2.8 ± 2.0 days/week). Participants reported an average of 7 h of sleep per day (6.9 ± 1.0 h/day) over the 8-week intervention. No snack group effect was detected for breakfast or sleep outcomes.

### 3.3. Compliance with Snack Consumption

Participants consumed their snacks on an average of 6 days per week (5.9 ± 1.7 days/week) over the 8-week intervention with no significant difference in overall compliance between the almond and cracker snack groups (*p* = 0.97). Average time of snack consumption was recorded as 11:00 am. In addition, data from 24-h recalls indicated the percentage of energy from fat and total alpha-tocopherol (i.e., nutrients rich in almonds) was greater in the almond group at the end of the 8-week intervention compared to the cracker group (group x time effect, *p* < 0.05) (Table 2). In general, the almond group reported greater total fat, MUFAs, oleic acid, and lower percentage of energy from carbohydrate compared to the cracker group (group effect, *p* < 0.05).

### 3.4. BM and Fat-Free Mass Increased over 8 Weeks in Both Snack Groups

Total BM significantly increased from baseline to week 4 and to week 8 of the intervention (time effect, *p* < 0.05, Table 3) while fat-free mass significantly increased from baseline to week 8 and week 4 to week 8 (time effect, *p* < 0.05, Table 3). Total fat mass, trunk fat mass, trunk fat-free mass, and waist circumference were not significantly different between baseline and week 8 (Table 3). All anthropometric outcomes were similar in both the almond and cracker groups at the end of the 8-week intervention.

### 3.5. Cardiovascular Outcomes Did Not Change Significantly over 8 Weeks in Both Snack Groups

Resting systolic blood pressure (SBP), diastolic blood pressure (DBP) and mean arterial pressure were not significantly different between baseline, week 4 and week 8 (Table 3). There was no difference in any of the blood pressure outcomes or RHI and AI between the almond and cracker groups at the end of the 8-week intervention (Table 3).

### 3.6. Almond Group Had a Smaller Decline in Fasting HDL Cholesterol over 8 Weeks Compared to the Cracker Group

**Figure 2:**
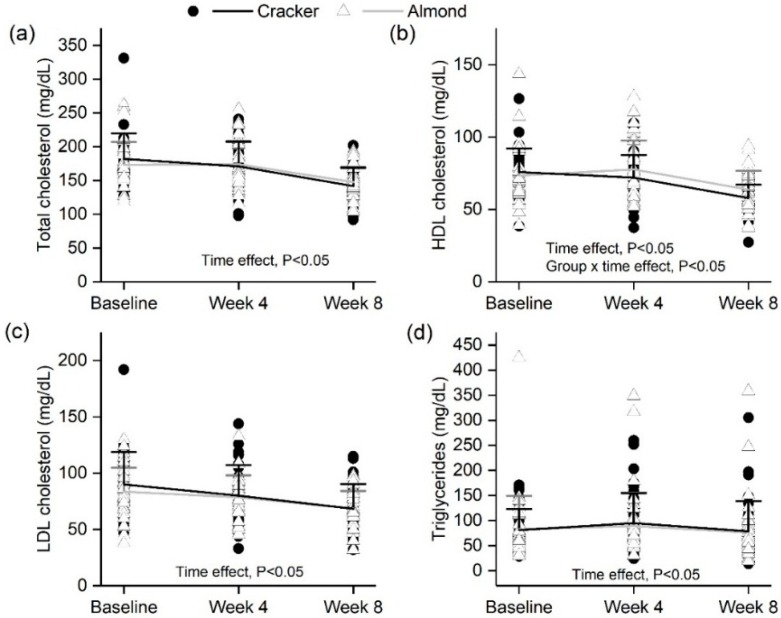
Fasting serum (**a**) total cholesterol, (**b**) HDL cholesterol (**c**) LDL cholesterol and (**d**) triglycerides profiles of the cracker and almond groups at baseline, week 4 and week 8 of the intervention. Values are individual data points representing each participant at baseline, week 4 and week 8. Means ± SDs of the 2 snack groups at baseline, week 4 and week 8 are also plotted. Analysis was conducted using a linear mixed effects model with week as within-subject factor and snack group as between-subject factor. Triglyceride values were transformed prior to analysis using Johnson’s family of transformations. Cracker: *n* = 35, Almond: *n* = 38. Total cholesterol time effect: baseline vs. week 8; week 4 vs. week 8, *p* < 0.05. HDL cholesterol time effect: baseline vs. week 8; week 4 vs. week 8, *p* < 0.05. LDL cholesterol time effect: baseline vs. week 4, *p* < 0.05; week 4 vs. week 8, *p* < 0.05. Triglycerides time effect: week 4 vs. week 8, *p* < 0.05.

Fasting serum total cholesterol and HDL cholesterol concentrations significantly decreased (time effect, *p* < 0.05) from baseline to week 8 and week 4 to week 8 of the intervention (Figure 2a,b). LDL cholesterol concentrations progressively decreased from baseline to week 4 to week 8 (*p* < 0.05, Figure 2c). In addition, triglyceride concentrations marginally decreased from week 4 to week 8 (time effect, *p* < 0.05) with no overall difference over the 8-week intervention (Figure 2d). However, the almond group had significantly lower decrease in HDL cholesterol over 8 weeks compared to the cracker group (13.5% vs. 24.5%, primary analysis: time x group effect, change analysis: group effect, *p* < 0.05, Figure 2b and Table S2) and a trend for smaller decrease in total cholesterol (primary analysis: time × group effect, *p* = 0.09). However, the secondary baseline-adjusted change analysis where week 4 was removed from consideration demonstrated a significant group effect, *p* = 0.04 (Table S2). Supplementary analyses indicate that overweight individuals in the cracker group had a greater decrease in HDL cholesterol compared to normal-weight individuals in the almond group (*p* < 0.05, Table S3). In addition, individuals with fasting glucose ≥100 mg/dL in both the cracker and almond groups had a greater decline in total, LDL and HDL cholesterol than individuals in the almond group with fasting glucose <100 mg/dL (*p* < 0.05, Table S5). Responder analysis also indicates that the cracker group had a higher proportion of individuals demonstrating a decline in LDL (regardless of magnitude) than the almond group (*p* < 0.05, Table S6).

### 3.7. Fasting Glucose Decreased but Insulin and GLP-1 Did Not Change Significantly over 8 Weeks in Both Snack Groups

**Figure 3:**
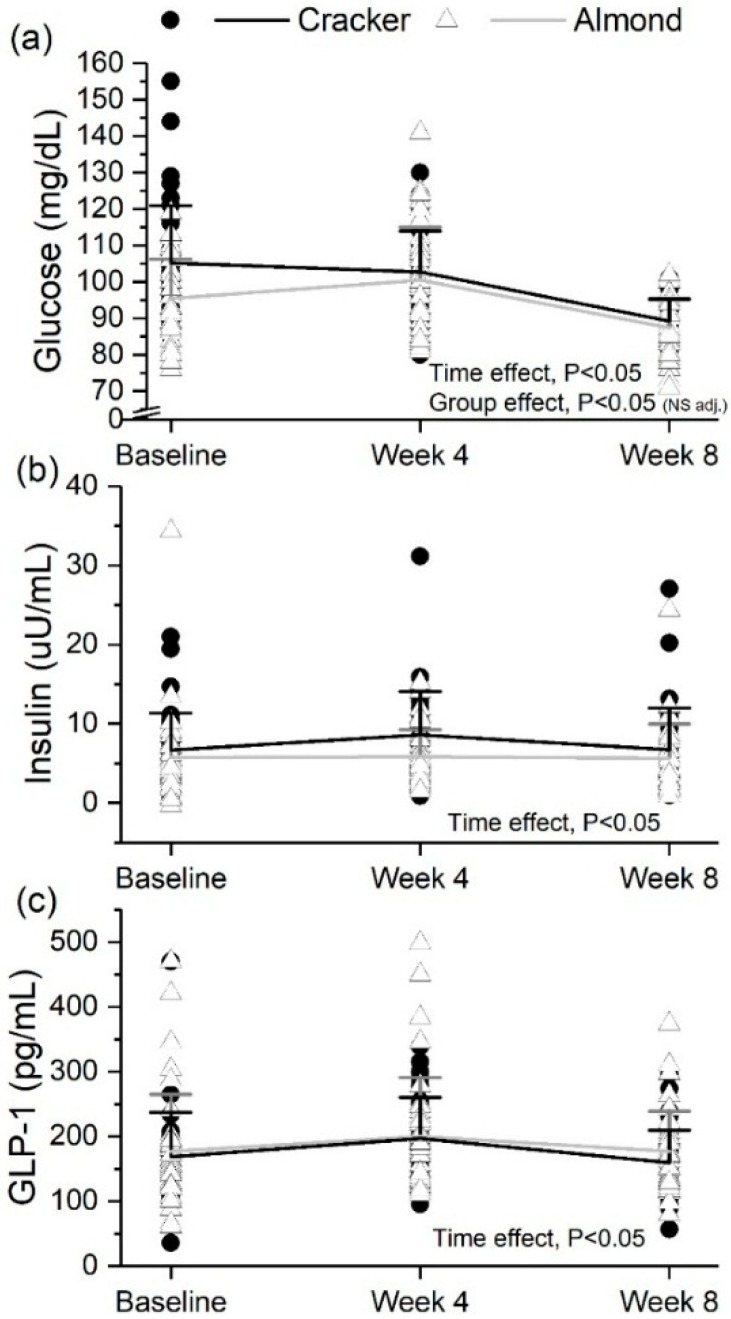
Fasting serum (**a**) glucose, (**b**) insulin and (**c**) GLP-1 profiles of the cracker and almond groups at baseline, week 4 and week 8 of the intervention. Values are individual data points representing each participant at baseline, week 4 and week 8. Means ± SDs of the 2 snack groups at baseline, week 4 and week 8 are also plotted. Analysis was conducted using a linear mixed effects model with week as within-subject factor and snack group as between-subject factor. Insulin and GLP-1 values were transformed prior to analysis using Johnson’s family of transformations. Cracker: *n* = 35, Almond: *n* = 38. Glucose time effect: baseline vs. week 8, *p* < 0.05; week 4 vs. week 8, *p* < 0.05. Insulin time effect: baseline vs. week 4, *p* < 0.05; week 4 vs. week 8, *p* < 0.05. GLP-1 time effect: baseline vs. week 4, *p* < 0.05; week 4 vs. week 8, *p* < 0.05. NS adj.: non-significant after baseline adjustment.

Fasting serum glucose concentrations significantly decreased from baseline to week 8 and week 4 to week 8 of the intervention (time effect, *p* < 0.05, Figure 3a). Fasting serum insulin concentrations significantly increased from baseline to week 4 but decreased from week 4 to week 8 of the intervention (time effect, *p* < 0.05, Figure 3b) with no significant change over the 8-week intervention. Fasting GLP-1 concentrations significantly increased from baseline to week 4; but, decreased from week 4 to week 8 of the intervention (time effect, *p* < 0.05, Figure 3c) with no significant change over 8 weeks. The cracker group had significantly higher glucose concentrations than the almond group (group effect, *p* < 0.05, Figure 3a), but after adjusting for baseline there was no significant group effect). Supplementary analyses indicate that individuals with total cholesterol ≥170 mg/dL in the cracker group had a greater decrease in fasting glucose compared to individuals with total cholesterol <170 mg/dL in the almond group (*p* < 0.05, Table S4).

### 3.8. Fasting β-Cell Function Increased over 8 Weeks in Both Snack Groups

HOMA-β, an index of fasting β-cell function [40], significantly increased from baseline to week 8 and week 4 to week 8 of the intervention (time effect, *p* < 0.05, Table 3). HOMA-IR, an index of insulin resistance in the fasting state [30], significantly increased from baseline to week 4 and decreased from week 4 to week 8 (time effect, *p* < 0.05, Table 3). QUICKI, an index of insulin sensitivity in the fasted state [30] significantly decreased from baseline to week 4, but increased from week 4 to week 8 of the intervention (time effect, *p* < 0.05, Table 3) with no significant change over the 8-week intervention. The cracker group had significantly higher HOMA-IR than the almond group (group effect, *p* < 0.05, Table 3), but after adjusting for baseline there were no significant group effects.

### 3.9. Almond Group Had Lower Glucose, Insulin and C-Peptide AUC_0–120min_ during the OGTT Compared to the Cracker Group at the End of the 8-Week Intervention

**Figure 4:**
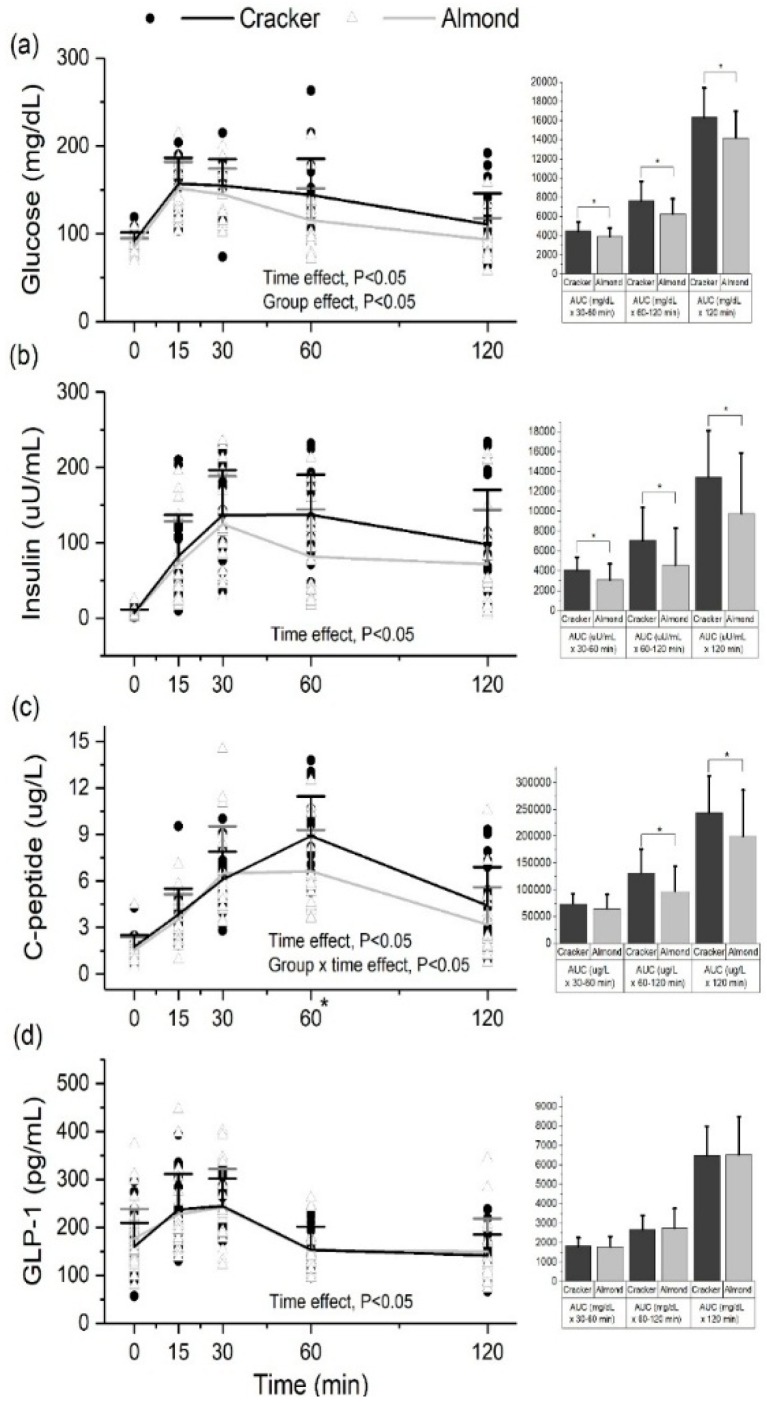
Serum (**a**) glucose, (**b**) insulin, (**c**) C-peptide and (**d**) GLP-1 profiles at 0, 15, 30, 60 and 120 min of an oral glucose tolerance test (OGTT) in the cracker and almond groups at week 8 of the intervention. Values are individual data points representing each participant at each time point. Means ± SDs of the 2 snack groups at each time point are also plotted. Analysis was conducted using a linear mixed effects model with time as within-subject factor and snack group as between-subject factor. Glucose, insulin, C-peptide and GLP-1 values were transformed prior to analysis using Johnson’s family of transformations. Area under the curves (Means ± SDs) are displayed adjacent to the respective OGTT profiles. Analysis was conducted using a Kruskal-Wallis test with snack group as between-subject factor. *, *p* < 0.05. Cracker: *n* = 20, Almond: *n* = 20. Glucose time effect: 15, 30, 60 vs. 0 min; 60, 120 vs. 15 min; 60, 120 vs. 30 min; 120 vs. 60 min, *p* < 0.05. Insulin time effect: 15, 30, 60, 120 vs. 0 min; 30, 60 vs. 15 min; 30 vs. 120 min, *p* < 0.05. C-peptide time effect: 15, 30, 60, 120 vs. 0 min; 30, 60 vs. 15 min; 60, 120 vs. 30 min; 120 vs. 60 min, *p* < 0.05. GLP-1-time effect: 15, 30, 120 vs. 0 min; 60, 120 vs.15 min; 60, 120 vs. 30 min, *p* < 0.05.

Serum glucose, insulin, C-peptide and GLP-1 concentrations significantly increased and subsequently decreased over the 2-h (120 min) period following consumption of a 75 g glucose drink (time effect, *p* < 0.05, Figure 4a–d). The cracker group had significantly higher serum glucose concentrations during the OGTT, glucose and insulin AUC_60–120min_, and glucose and insulin AUC_0–120min_ compared to the almond group (group effect, *p* < 0.05) (Figure 4a,b). The cracker group also had significantly higher serum C-peptide concentrations at 60 min (group × time effect, *p* < 0.05), C-peptide AUC_60–120 min_ and C-peptide AUC_0–120 min_ compared to the almond group (group effect, *p* < 0.05, Figure 4c).

### 3.10. Almond Group Had Higher Insulin Sensitivity during the OGTT Compared to the Cracker Group at the End of the 8-Week Intervention

**Figure 5:**
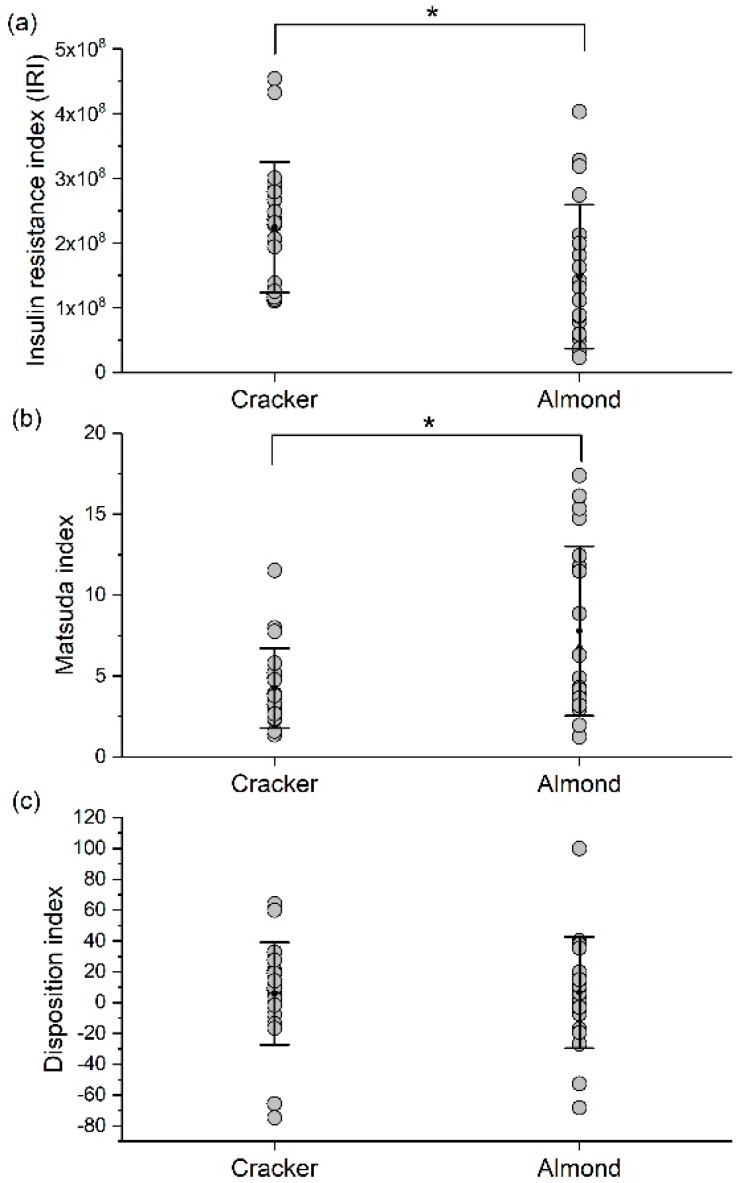
(**a**) Insulin resistance index (IRI), (**b**) Matsuda index and (**c**) disposition index during an OGTT in the cracker and almond groups at week 8 of the intervention. Values are individual data points representing each participant at each time point. Means ± SDs of the 2 snack groups at each time point are also plotted. Analysis for indices was conducted using a Kruskal-Wallis test with snack group as between-subject factor. *, *p* < 0.05. Cracker: *n* = 20, Almond: *n* = 20.

The IRI was significantly lower (34%) and Matsuda index was significantly higher (82%) in the almond group compared to the cracker group (group effect, *p* < 0.05, Figure 5a,b). The disposition index was not significantly different between the cracker and almond groups (Figure 5c). Supplementary analyses indicate that normal-weight individuals in the almond group had a higher Matsuda Index than obese individuals in the cracker group (*p* < 0.05, Table S8).

### 3.11. 24-h Free-Living Appetite Ratings Did Not Change Significantly over 8 Weeks in Both Snack Groups

Mean 24-h hunger, desire to eat, and prospective consumption ratings significantly decreased from baseline to week 4. All but prospective consumption ratings increased from week 4 to week 8 with no overall difference between baseline and week 8 of the intervention (time effect, *p* < 0.05, Table 3). Twenty-four fullness ratings did not significantly differ over the 8-week intervention (Table 3).

### 3.12. Snack Palatability and Acceptance Ratings Declined over 8 Weeks but Almonds Had Higher Acceptance Than Crackers

Palatability ratings significantly decreased after the intervention from moderately palatable to neutral palatability (−18.6 ± 40.8 gLMS units; time effect, *p* < 0.05) and acceptance ratings significantly decreased from “I like this and would eat it now and then” to “I would eat this if available but would not go out of my way” (−0.8 ± 2.0 FACT scale units). However, almonds had significantly higher acceptance overall compared to crackers (5.4 ± 1.7 vs. 4.6 ± 1.6 FACT scale units, group effect, *p* < 0.05).

### 3.13. Activity Energy Expenditure Decreased on the Weekends over the 8-Week Intervention

Mean activity energy expenditure decreased over the weekends (2.2 ± 1.8 (baseline) vs. 1.08 ± 1.04 (week 8) kcal/minute) and was lower compared to weekdays (2.1 ± 1.8 (baseline) vs. 2.01 ± 1.0 (week 8) kcal/minute, time x weekday/weekend effect, *p* < 0.05) at the end of the 8-week intervention. There was no significant difference in activity energy expenditure between the almond and cracker groups.

## 4. Discussion

The present study demonstrated that almond snacking was associated with greater glucose tolerance and whole-body insulin sensitivity (assessed with indices of postprandial state) at 8 weeks and smaller decline in HDL cholesterol but similar decline in fasting glucose as the cracker group in spite of minimal gains in body mass over 8 weeks. It is possible that in young adults with no cardiometabolic disorders, the benefits of chronic almond consumption may manifest as acute improvements in glucose tolerance. Postprandial glucose may serve as a better marker for detecting early perturbations in glucose metabolism in nondiabetic individuals than fasting glucose as individuals spend most of the day in the postprandial or post-absorptive state [41]. Postprandial (2 h) glucose is also a strong predictor of mortality and cardiometabolic disorders [41]. Acute feeding studies in healthy adults have demonstrated benefits of almonds when eaten with meals on postprandial glucose [6,42] and insulin [42] responses. Almond consumption also has second-meal effects on glycemic control, but was observed previously only in individuals with impaired glucose tolerance [5]. The second-meal effect is a phenomenon where a prior meal attenuates postprandial blood glucose responses to a subsequent meal [43,44]. Studies investigating the chronic effects of almond snacking on postprandial glycemic control are scarce, short-term (i.e., 4 weeks), conducted in at-risk individuals [17], or T2DM individuals [45], and have not measured a small suite of glucoregulatory hormones simultaneously. The present study expands the evidence on these effects in young adults. The unique nutrient composition of almonds, which are rich in unsaturated fats, fiber, and polyphenols and low in simple carbohydrates, has a moderating effect on postprandial glycemia [5,6,42] suppressing glucose excursions, which may contribute to benefits with long-term consumption in the dynamic postprandial state.

The positive relationship between adiponectin and insulin sensitivity is well established [46]. The present study supports this contention as the integrated adiponectin response to OGTT was positively correlated with the Matsuda index (index of insulin sensitivity, supplementary analyses) suggesting that improvements in glucose tolerance are at least partially mediated by adiponectin. However, almond snacking did not differentially influence the stimulation of GLP-1 during the OGTT in this study, indicating that the glucoregulatory benefits were possibly independent of incretin response.

Almond snacking for 8 weeks had a marginally protective effect on HDL cholesterol by ameliorating the decrease (13.5%) compared to the cracker group (24.5%) but had similar beneficial decreases in LDL cholesterol as the cracker group. Although there was a trend towards a smaller decline in total cholesterol with almond snacking as well, the change in total cholesterol was largely driven by the change in HDL cholesterol. In contrast, a recent systematic review observed a reduction in total and LDL cholesterol but no change in HDL cholesterol with almond consumption [47]. However, the review incorporated studies conducted on participants of a much greater median age. In a very recent and novel study published after the review, almond (43 g/day) consumption improved α-1 HDL and α-1/pre–β-1 HDL subspecies compared to the control in individuals of normal weight albeit elevated LDL cholesterol [48]. These favorable changes in HDL subspecies may not be captured in studies assessing only total HDL cholesterol, for example, the present study and those included in the systematic review [47]. Since α-1 HDL is a better (negative) predictor of coronary heart disease than total HDL cholesterol [49], measuring HDL subspecies in future studies is important. On the other hand, almond and cracker snacking resulted in similar declines in fasting glucose and improvements in β-cell function (HOMA-β) over the 8-week intervention. The decline in fasting glucose levels over 8 weeks with both almond and cracker snacking is likely due to increased frequency of breakfast/morning snack consumption (and not increased insulin/incretin response) since skipping breakfast is associated with higher fasting blood glucose [50,51]. However, the lack of a “no morning snack” group is an important limitation that precludes assessment of breakfast skipping physiological responses.

The 8-week intervention resulted in minimal (0.8 kg) body mass gain in free-living and predominantly breakfast skipping college students regardless of snack group. The decrease in activity energy expenditure on the weekends may have contributed to body mass gain. Several studies have indicated significant positive associations between body mass gain in college and a decrease in physical activity, in some cases, despite decreased energy intake [52,53,54]. The typical mass gain for freshmen students is 1–3 kg over the academic year [18,19], amounting to 0.1 kg/wk in the worst-case scenario, which is the same rate of gain observed in the present study, suggesting intervention-associated gains were not exceptional but expected. Although the mass gained was mostly fat-free mass (0.6 kg), the limitations of bioelectrical impedance analysis in accurately assessing body composition [55], particularly in individuals with obesity [56], and the limitations of accelerometers in assessing activity during strength training [57] should be considered while interpreting this finding. Our findings are in contrast with the preponderance of nut and body mass literature which suggests that nut consumption does not lead to significant gains in body mass [58], likely due to the confounding effects of physical activity. Supplementary analyses further suggest that overweight and obesity, and higher fasting glucose and total cholesterol levels may differentially influence the response to a high-fat (almond) versus a high-carbohydrate (cracker) snack. Future studies need to be powered adequately to confirm the reliability of these responses.

Although the acute benefits of almond consumption on appetite are well documented [17,59,60], the long-term effects of almond snacking in young adults are unclear. The present study demonstrated a similar decline in subjective hunger and desire to eat ratings at 4 weeks with almond and cracker consumption, and a subsequent increase at 8 weeks resulting in no significant change over 8 weeks. Interestingly, this temporal pattern of appetite ratings corresponded with an increase in satiety regulating gut peptide, GLP-1, at week 4 and decrease at week 8 with no significant change over the 8-week intervention as well. Hence, while the macronutrient composition of a food contributes to the regulation of appetite acutely [61], the differential appetitive effects may not be sustained in chronic studies.

The decline in almond and cracker palatability and acceptance ratings from a moderate state at baseline to a neutral state at the end of the intervention could be a result of monotony effects arising from repeated daily consumption [62]. Repeated nut consumption has dose-dependent monotony effects with higher doses (60 g/day) associated with decreased acceptance compared to lower doses (30 g/day) [63]. One study found that long-term (12-week) almond consumption under energy restriction conditions did not decrease palatability and acceptance in compliant individuals [64]. However, the study individualized the almond dose (15% of energy-restricted diet) [64] compared to the fixed 56.7 g/day dose in the present study. Nevertheless, almonds had significantly higher acceptance throughout the study compared to crackers, suggesting that repeated consumption of a nutritious almond snack is well accepted over a typical refined carbohydrate snack in this population.

An important strength of the study lies in the extremely high compliance of snack consumption, likely resulting from the deployment of peer study researchers used to visually ensure daily participant consumption. To our knowledge, this is the first almond consumption study to recruit first-year college students, the majority of whom were breakfast skippers. A limitation of the study was not conducting the same 5-time point OGTT prior to the intervention, as performed at 8 weeks, to allow for a pre-post intervention assessment. However, there were no differences in baseline fasting insulin sensitivity between groups. In addition, we performed a modified OGTT measuring blood glucose by finger sticks at 0, 60 and 120 min. These data demonstrated no significant group effect on glucose AUC_0–120min_ suggesting that the groups were similar with respect to their glucose tolerance status at the onset of the intervention (data not shown). Another limitation was the 7-day spring break that immediately followed the mid-point of the intervention; however, the lack of remarkable differences at week 4 that were ultimately captured at week 8, implies that the impacts of such an interruption at the mid-point were not profound. 

## 5. Conclusions

Almond consumption (56.7 g/2 oz.) in an 8-week, free-living environment, marginally ameliorated the decline in HDL cholesterol and resulted in greater postprandial glucose tolerance and insulin sensitivity than a cracker snack despite expected minimal gain in body mass. In general, incorporating a morning snack in the dietary regimen of first-year college students, a group which is susceptible to poor lifestyle habits as they transition to independence, reduced fasting glucose and cholesterol. However, cardiometabolic risk factors may differentially influence the response to cracker vs. almond consumption. The physiological and clinical significance of these changes have yet to be examined and may be more pronounced in youth with or at high risk for cardiometabolic disorders.

## Figures and Tables

**Table 1:** Baseline demographic and clinical characteristics of participants in the cracker and almond groups.

Characteristics	Cracker (*n* = 35)	Almond ^a^ (*n* = 38)
Sex, *n* (%)		
Male	16 (45.7)	16 (42.1)
Female	19 (54.3)	22 (57.9)
Age, years (range)		
18	34 (97)	38 (100)
19	1 (3)	0 (0)
Race/Ethnicity, *n* (%)		
Hispanic	16 (45.7)	15 (39.5)
Asian/Pacific Islander	13 (37.1)	14 (36.8)
African American	2 (5.7)	5 (13.2)
Caucasian White	4 (11.4)	4 (10.5)
BMI, kg/m^2^	25.3 ± 4.5	25.6 ± 5.0
BMI Category, *n* (%)		
Normal weight (5th–85th percentile)	22 (63)	28 (74)
Overweight (85th–95th percentile)	8 (23)	6 (16)
Obese (≥95th percentile)	5 (14)	4 (11) *
Waist circumference, cm		
≥94 (M), ≥80 (F)	9 (26)	10 (26)
<94 (M), <80 (F)	26 (74)	28 (74)
Fasting glucose **		
<100 mg/dL	13 (37)	23 (62)
≥100 mg/dL	22 (63)	14 (38)
HOMA-IR		
<1.0	9 (26)	18 (49)
1.0–2.0	15 (43)	11 (30)
≥2.0	11 (31)	8 (22)
Total cholesterol		
<170 mg/dL	12 (34)	21 (57)
≥170 mg/dL	23 (66)	16 (43)
LDL cholesterol		
<100 mg/dL	23 (66)	29 (78)
≥100 mg/dL	12 (34)	8 (22)
HDL cholesterol		
<45 mg/dL	1 (3)	1 (3)
≥45 mg/dL	34 (97)	36 (97)
Triglycerides		
<150 mg/dL	32 (91)	36 (97)
≥150 mg/dL	3 (9)	1 (3)
Systolic BP		
<130 mmHg	33 (94)	32 (84)
≥130 mmHg	2 (6)	6 (16)
Diastolic BP		
<85 mmHg	35 (100)	37 (97)
≥85 mmHg	0 (0)	1 (3)
IDF Metabolic syndrome risk factors		
0	10 (29)	13 (34)
1	14 (40)	18 (47)
2	10 (29)	6 (16)
3	1 (3)	1 (3)

Values are *n* (%). ^a^, Lipid profile, glucose, HOMA-IR reported for 37 participants. * two individuals with BMI ≥99th percentile. ** Chi-squared test, *p* < 0.05. IDF, International Diabetes Federation. Glucose: 100 mg/dL = 5.6 mmol/L. Total cholesterol: 170 mg/dL = 4.4 mmol/L. LDL cholesterol: 100 mg/dL = 2.6 mmol/L. HDL cholesterol: 45 mg/dL = 1.2 mmol/L. Triglycerides: 150 mg/dL = 1.7 mmol/L.

**Table 2:** Nutrient intakes in the cracker and almond groups at baseline, week 4 and week 8 of the study.

Nutrients	Cracker (*n* = 35)			Almond (*n* = 38)			*p*-Values		
	Baseline	Week 4	Week 8	Baseline	Week 4	Week 8	Group	Week	Group × Week
Energy, kcal	1618.3 ± 524.1	1650.4 ± 603.9	1624.4 ± 470.2	1677.6 ± 714.4	1774.3 ± 772.6	1722.2 ± 603.4	0.366	0.793	0.942
Carbohydrate, g/day	205.1 ± 12.9	210.9 ± 78.3	217.1 ± 70.6	188.9 ± 86.6	208.9 ± 109.3	183.7 ± 69.7	0.214	0.523	0.441
Carbohydrate, % energy	52 ± 1.8	51.8 ± 10.1	53.7 ± 9.9	45.5 ± 10.4	46.3 ± 11.2	42.7 ± 10.2	**<0.001**	0.834	0.158
Fat ^Ɨ^, g/day	62.2 ± 5.4	63.3 ± 32.7	61 ± 23.1	65.9 ± 35	74.5 ± 41.8	79.9 ± 32.6	**0.047**	0.261	0.342
Fat, % energy	33.3 ± 1.4	33.5 ± 7.6	33.5 ± 7.2 ^a^	34.8 ± 8.4	38 ± 9.7	41.7 ± 8.1	**0.002**	**0.013** ^d^	**0.021**
Protein ^Ɨ^, g/day	64.2 ± 7.2	62.6 ± 34.9	56.3 ± 26.6	83.6 ± 51.1	73 ± 35.5	72 ± 34.2	0.014 ^(NS adj.)^	0.365	0.823
Protein ^Ɨ^, % energy	15.5 ± 1.1	15.5 ± 6.5	13.9 ± 5.4	19.9 ± 8.3	17.2 ± 7	16.9 ± 6.5	0.006 ^(NS adj.)^	**0.04** ^d^	0.504
Dietary fiber, g/day	13.6 ± 1	15.2 ± 7.3	13.9 ± 7.3	12.5 ± 6.3	16.2 ± 8.2	13.8 ± 6.2	0.979	**0.016** ^b^	0.542
Total MUFAs ^Ɨ^, g/day	20.9 ± 2.2	22.9 ± 12	22 ± 8.6	23.3 ± 14.7	29.3 ± 17.1	31.8 ± 14.1	**0.013**	**0.014** ^d^	0.224
Total PUFAs ^Ɨ^, g/day	17.2 ± 1.7	15.8 ± 9.2	15.2 ± 7.7	16.3 ± 10.1	17.7 ± 10.6	20.1 ± 9.9	0.191	0.511	0.207
Total SFAs ^Ɨ^, g/day	19.1 ± 1.8	19.7 ± 12.1	19.2 ± 9.8	21.1 ± 12.1	21.8 ± 15.3	22.2 ± 11.7	0.357	0.909	0.963
Oleic acid ^Ɨ^, g/day	19.5 ± 2	21.4 ± 11.2	20.7 ± 8.1	21.5 ± 13.6	27.6 ± 16.5	30.1 ± 13.6	**0.013**	**0.009** ^bc^	0.223
Linoleic acid ^Ɨ^, g/day	15.2 ± 1.6	14.1 ± 8.3	13.7 ± 7	14.1 ± 8.9	15.8 ± 9.7	18.1 ± 8.9	0.205	0.339	0.189
Total alpha-tocopherol, mg/day	6.5 ± 0.5	7.2 ± 5.2 ^a^	6.5 ± 5.4 ^a^	6.3 ± 3.7	13.2 ± 9.7	12.6 ± 8.4	**<0.001**	**0.001**	**0.005**
Magnesium, mg/day	220.9 ± 16.9	219.4 ± 81.8 ^a^	211.1 ± 86	230.6 ± 119.4	305.6 ± 133	261.7 ± 106.6	**0.01**	**0.020** ^b^	**0.027**
Selenium ^Ɨ^, mg/day	88.4 ± 9.7	83.7 ± 39.8	79.3 ± 36.2	119.3 ± 70	95.5 ± 57.3	95.9 ± 50.3	0.040 ^(NS adj.)^	**0.046**	0.394
Phosphorous ^Ɨ^, mg/day	998.4 ± 86.9	1019 ± 432.9	953.8 ± 377.1	1167.7 ± 572.3	1234.3 ± 538.8	1105.7 ± 478.8	0.056	0.298	0.893
Sodium ^Ɨ^, mg/day	2962.3 ± 248.2	2987 ± 1226.7	2780.7 ± 1304.3	3210.4 ± 1599.3	3048.6 ± 1539.7	2861.3 ± 1357.9	0.6879	0.403	0.764
Peanuts, tree nuts, and seeds; excludes coconut (oz. eq.)	0.1 ± 0	0.5 ± 1.2 ^a^	0.5 ± 1.5 ^a^	0.1 ± 0 ^bd^	2.2 ± 2.5	2.1 ± 2.3	-	-	**<0.001** *

Values are means ± SDs. Analysis was performed using a linear mixed effects model with week as within-subject factor and snack group as between-subject factor. *, non-parametric Kruskal-Wallis test with Dunn joint ranks method for pairwise comparisons for snack × week categories. ^Ɨ^, values transformed prior to analysis using Johnson’s family of transformations, NS adj., non-significant after baseline adjustment Values in bold are *p* < 0.05. -, not assessed. ^a^, cracker vs. almond *p* < 0.05; ^b^, baseline vs. week 4, *p* < 0.05; ^c^, week 4 vs. week 8, *p* < 0.05; ^d^, baseline vs. week 8, *p* < 0.05.

**Table 3:** Anthropometric, clinical, cardiovascular and appetite outcomes in the cracker and almond groups at baseline, week 4 and week 8 of the study.

Characteristic	Cracker (*n* = 35)			Almond (*n* = 38)			*p*-Values		
	Baseline	Week 4	Week 8	Baseline	Week 4	Week 8	Group	Week	Group × Week
Body mass ^Ɨ^, kg	71.3 ± 15.1	71.3 ± 14.7	71.9 ± 15	71.5 ± 18.6	72 ± 18.8	72.5 ± 19	0.781	**<0.001** ^abc^	0.386
Total fat ^Ɨ^, %	27.9 ± 9	27.5 ± 9.2	27.8 ± 9.4	26.9 ± 9.5	27.2 ± 9.6	27 ± 9.4	0.926	0.461	0.856
Total fat mass, kg	19.9 ± 10.5	20 ± 10.1	20.5 ± 10.2	19.8 ± 11.2	20.1 ± 11.1	20.1 ± 11.1	0.969	0.202	0.239
Total fat-free mass ^Ɨ^, kg	50.9 ± 10	51.2 ± 9.9	51.4 ± 10	51.7 ± 12.7	51.9 ± 12.7	52.4 ± 13	0.882	**0.011** ^bc^	0.233
Trunk fat ^Ɨ^, %	25.6 ± 9.1	25.7 ± 9.4	25.9 ± 9.5	24 ± 10.8	24.8 ± 10.9	24.2 ± 10.3	0.465	0.529	0.634
Trunk fat mass ^Ɨ^, kg	10.2 ± 5.4	10.2 ± 5.5	10.9 ± 6.4	9.7 ± 6.7	9.9 ± 6.4	9.9 ± 6.2	0.462	0.539	0.747
Trunk fat-free mass, kg	28.6 ± 5.4	27.8 ± 6.9	28.7 ± 5.5	28.9 ± 6.5	28.7 ± 6.4	29.1 ± 6.7	0.716	0.223	0.741
Waist circumference ^Ɨ^, cm	80.4 ± 10.3	80.5 ± 10.1	80.6 ± 10.5	80.5 ± 11.1	81.3 ± 11.3 ^b^	80.2 ± 11.2	0.942	0.081	**0.024**
HOMA-IR ^Ɨ^	1.8 ± 1.3	2.2 ± 1.4	1.5 ± 1.2	1.4 ± 1.4	1.5 ± 1	1.2 ± 1.1	0.040 ^(NS adj.)^	**<0.001** ^ab^	0.369
HOMA-β ^Ɨ^, %	58.7 ± 44.7	80.9 ± 50.6	89.5 ± 61.3	68 ± 60	61.4 ± 40.2	84.3 ± 53.9	0.495	**<0.001** ^bc^	0.125
QUICKI ^Ɨ^	0.38 ± 0.09	0.35 ± 0.04	0.38 ± 0.05	0.39 ± 0.06	0.37 ± 0.04	0.39 ± 0.05	0.052	**<0.001** ^ab^	0.399
Resting systolic blood pressure, mmHg									
Systolic BP	107.8 ± 15.4	106.2 ± 14.6	108.5 ± 12.5	112.4 ± 14.4	109.9 ± 14.3	108.2 ± 14.9	0.366	0.285	0.175
Diastolic BP	67.6 ± 6.5	68.6 ± 6.4	68.4 ± 6.9	67.9 ± 8.5	67.1 ± 6.4	67.7 ± 7.2	0.625	0.927	0.502
Mean arterial pressure	81 ± 8.6	81.1 ± 8.3	81.8 ± 8.1	82.7 ± 8.6	81.3 ± 8.1	81.2 ± 8.1	0.797	0.727	0.321
Reactive hyperemia index (RHI)	-	-	2.0 ± 0.7	-	-	1.9 ± 0.6	0.558 *	-	-
Augmentation index (AI)	-	-	−8.7 ± 5.7	-	-	−6.5 ± 10.8	0.193 *	-	-
Appetite ratings, mm									
Hunger	31.1 ± 13.3	27.4 ± 14.7	30.5 ± 13.9	34.7 ± 13.7	29.2 ± 15.5	34.5 ± 12.6	0.284	**0.014** ^ab^	0.721
Fullness	48.4 ± 16	48.5 ± 17.1	48.6 ± 16.8	47.5 ± 16.5	52.5 ± 15.5	48.6 ± 14	0.731	0.343	0.347
Desire to eat	27.8 ± 13.2	23 ± 12.5	27 ± 15.4	32.6 ± 13.2	26 ± 14.8	30 ± 13.6	0.202	**0.001** ^ab^	0.807
Prospective consumption	29.5 ± 12.6	26.4 ± 14.3	29.9 ± 15.8	36.4 ± 17.9	30.1 ± 15.3	33 ± 13.8	0.147	**0.011** ^a^	0.469

Values are means ± SDs. Analysis was performed using a linear mixed effects model with week as within-subject factor and snack group as between-subject factor. *, one-way ANOVA with snack group as between-subject factor. ^Ɨ^, values transformed prior to analysis using Johnson’s family of transformations, ^NS adj.^, non-significant after baseline adjustment. Values in bold are *p* < 0.05. -, not assessed. ^a^, baseline vs. week 4, *p* < 0.05; ^b^, week 4 vs. week 8, *p* < 0.05; ^c^, baseline vs. week 8, *p* < 0.05. HOMA- homeostasis model assessment, IR-insulin resistance, QUICKI- quantitative insulin sensitivity check.

## Supplementary Materials

The following are available online at http://www.mdpi.com/2072-6643/10/8/960/s1, Table S1: Nutrient composition of the cracker and almond snacks, Table S2: Table Anthropometric, clinical, cardiovascular and appetite outcome changes over 8 weeks (baseline-week 8) by snack group, Table S3: Anthropometric, clinical, cardiovascular and appetite outcome changes over 8 weeks (baseline-week 8) by snack group and baseline BMI category, Table S4: Anthropometric, clinical, cardiovascular and appetite outcome changes over 8 weeks (baseline-week 8) by snack group and baseline fasting total cholesterol category, Table S5: Anthropometric, clinical, cardiovascular and appetite outcome changes over 8 weeks (baseline-week 8) by snack group and baseline fasting glucose category, Table S6: Non-responders and responders of specific outcomes in the cracker and almond groups, Table S7: *p*-values for Figure 2, Figure 3, Figure 4 and Figure 5, S1 and S2 main effects, Table S8: OGTT area under the curves and indices by snack group and baseline BMI category, Figure S1: Fasting (a) serum NEFAs, (b) serum adiponectin and (c) plasma leptin profiles of the cracker and almond groups at baseline, week 4 and week 8 of the intervention. Figure S2: Serum (a) glucagon, (b) adiponectin, (c) and NEFAs profiles at 0, 15, 30, 60 and 120 min of an OGTT in the cracker and almond groups at week 8 of the intervention. Figure S3: Variable importance projection (VIP) score vs. coefficient plot from the pruned partial least squares discriminant analyses (PLS-DA). Figure S4: Principal component analysis (A) score plot of participants and (B) loading plot of variables—i.e., ∆glucose, ∆total cholesterol, ∆LDL cholesterol and ∆HDL cholesterol identified by PLS-DA as most discriminatory between the snack groups over the 8-week intervention. Figure S5: Principal component analysis (A) score plot of participants and (B) loading plot of variables i.e., adiponectin AUC, C-peptide AUC, insulin AUC, glucose AUC, IRI, QUICKI, HOMA-IR and Matsuda index identified by PLS-DA as most discriminatory between the snack groups during the OGTT.
