# Supplementary material for: Glucoregulatory and Cardiometabolic Profiles of Almond vs. Cracker Snacking for 8 Weeks in Young Adults: A Randomized Controlled Trial
Source: Nutrients. 2018 Jul 25;10(8):960. doi: 10.3390/nu10080960 (PMC6115851; doi:10.3390/nu10080960)
Supplement: Supplementary file 1 [file nutrients-10-00960-s001.pdf]

Table S1: Nutrient composition of cracker and almond snacks

	Cracker*	Almond**
Weight, g	77.5 g (5 sheets)	56.7 g (2 oz.)
Energy, kcal	338	364
Carbohydrate, g (kcal)	62.5	12.6
Dietary fiber, g	2.5	8.1
Protein, g (kcal)	5	11.4
Total fat, g (kcal)	7.5	29.9
Total MUFA, g	1.25	19.1
Total PUFA, g	3.75	7.1
Total saturated fat, g	-	2.3

* Nutrient composition of Nabisco honey maid honey grahams crackers was obtained from the USDA food composition database.

** Nutrient composition of natural, whole, unsalted, nonpareil and dry roasted almonds was provided by the Almond Board of California

Table S2: Anthropometric, clinical, cardiovascular and appetite outcome changes over 8 weeks (baseline-week 8) by snack group

	Cracker		Almond		P-value
	Mean±SD	95% CI	Mean±SD	95% CI	Group
ΔBody mass, kg	-1.5±0.6	(-2.6,-0.3)	-2.1±0.5	(-3.2, -1)	0.4231
ΔFat mass, kg	-1.4±1	(-3.3,0.5)	-0.6±0.9	(-2.5, 1.2)	0.5702
ΔFat free mass, kg	-1.2±0.6	(-2.3,0)	-1.5±0.6	(-2.6, -0.3)	0.7322
ΔWaist circumference, cm	-0.1±0.1	(-0.4,0.2)	0.1±0.1	(-0.2, 0.4)	0.2822
ΔSystolic blood pressure, mmHg	0.3±1.8	(-3.3,3.8)	3.3±1.7	(-0.1, 6.7)	0.2189
ΔDiastolic blood pressure, mmHg	-0.8±1	(-2.8,1.3)	0.2±1	(-1.8, 2.1)	0.523
ΔTotal cholesterol, mg/dl	38.8±3.5	(31.9,45.8)	28.8±3.4	(22.1, 35.5)	**0.0438**
ΔLDL cholesterol, mg/dl	20.8±2.4	(16,25.7)	16.6±2.3	(12, 21.2)	0.2083
ΔHDL cholesterol, mg/dl	17.7±1.4	(14.9,20.5)	10.7±1.3	(8, 13.4)	**0.0006**
ΔTriglycerides, mg/dl	3.6±8.7	(-13.8,21.1)	5.6±8.4	(-11.2, 22.3)	0.8727
ΔGlucose, mg/dl	12±1.1	(9.8,14.3)	11.6±1.1	(9.5, 13.8)	0.8161
ΔInsulin, uU/ml	-0.2±0.6	(-1.3,1)	0.3±0.6	(-0.8, 1.4)	0.5794
ΔGLP-1, pg/ml	11.3±8.1	(-4.9,27.6)	-2.1±7.5	(-17, 12.8)	0.2285

ΔLeptin, ng/ml	-4±2	(-8,-0.04)	-0.8±1.9	(-4.5, 3)	0.2411
ΔNEFAs, mEq/l	0.1±0.02	(0.1,0.2)	0.1±0.02	(0.1, 0.2)	0.8292
ΔAdiponectin, ug/ml	1±0.7	(-0.3, 2.4)	0.8±0.6	(-0.4, 2)	0.7889
ΔQUICKI	0.01±0.01	(-0.01,0.02)	-0.01±0.01	(-0.02, 0.01)	0.3307
ΔHOMA-β	-28.1±8.2	(-44.5,-11.8)	-15.1±7.9	(-30.7, 0.6)	0.2539
ΔHOMA-IR	0.2±0.1	(-0.1,0.5)	0.2±0.1	(-0.03, 0.5)	0.8496
ΔHunger, mm	2.1±2	(-2,6.1)	-0.7±1.9	(-4.5, 3.2)	0.33
ΔFullness, mm	-0.7±2.1	(-4.9,3.6)	-0.9±2	(-4.9, 3.1)	0.9359
ΔDesire to eat, mm	2.1±2.1	(-2.1,6.4)	1.6±2	(-2.4, 5.6)	0.8637
ΔProspective consumption, mm	1.4±2.1	(-2.7,5.6)	2±2	(-1.9, 5.9)	0.8301

Values are baseline-adjusted least square means ± SEs of change (Δ, baseline-week 8) in outcomes. Positive values indicate decline over 8 weeks whereas negative values indicate increase over 8 weeks. Analysis was performed using a one-way ANOVA with snack (almond or cracker) as between subject factor and baseline value as covariate. Values in bold are P<0.05. HOMA- homeostasis model assessment, IR-insulin resistance, QUICKI- quantitative insulin sensitivity check

Table S3: Anthropometric, clinical, cardiovascular and appetite outcome changes over 8 weeks (baseline-week 8) by snack group and baseline BMI category

	Normal weight				Overweight				Obese				P-value
	Cracker		Almond		Cracker		Almond		Cracker		Almond		(Group x BMI)
	Mean±SD	N	Mean±SD	N	Mean±SD	N	Mean±SD	N	Mean±SD	N	Mean±SD	N	
ΔBody mass, kg	-1.7±2.9	22	-2.1±3.8	28	-1.2±1.8	8	-0.4±2	6	-0.9±3.1	5	-4.4±6	4	0.5223
ΔFat mass, kg	-1.4±8.6	22	-0.6±2.9	28	-0.4±2.6	8	-1±3.1	6	-2.8±2.9	5	-0.3±12.2	4	0.8101
ΔFat free mass, kg	-2±3.6 (32.3)	22	-1.5±2.6 (33.4)	28	-0.7±2.9 (39.8)	8	0.6±2.3 (48.2)	6	1.9±0.8	5	-4.2±8.3	4	**0.0467**
ΔWaist circumference, cm	-0.1±0.8	22	0.1±0.8	28	-0.2±0.8	8	0.4±0.4	6	0±1.6 (62.9)	5	0.1±0.1 (33)	4	0.5286
ΔSystolic blood pressure, mmHg	-3.9±13.6	22	3.3±7.7	28	4.1±6.1	8	1.6±5.7	6	6±7.7	5	14.4±30.6	4	0.1874
ΔDiastolic blood pressure, mmHg	-1.3±6.4	22	0.4±7.3	28	1.1±3.6	8	4.3±9.6	6	-1.6±8.1	5	-6.9±7.2	4	0.3907
ΔTotal cholesterol, mg/dl	38.3±33.9	22	23.7±26.7	27	51±23.4	7	36.5±31.2	6	45.6±26.1	5	25±26.2	4	0.0991
ΔLDL cholesterol, mg/dl	23.5±22.7	22	13.3±18.9	27	21.7±8.3	7	17.7±15	6	20.8±12.4	5	20.8±13.5	4	0.4122
ΔHDL cholesterol, mg/dl	15.2±11.8 (39.5)	22	9.2±12.6 (26)a	27	28.9±13 (58)b	7	11.3±12.6 (34)	6	18.8±13 (44.9)	5	12.9±6.3 (37.6)	4	**0.0072**
ΔTriglycerides, mg/dl	-2.3±49.5	22	6±35.6	27	2.6±46	7	37±83.4	6	30.4±36.4	5	-43.3±124.5	4	0.7552

ΔGlucose, mg/dl	14.1±9	22	8.4±10.6	27	18.1±22.2	7	6.3±11.1	6	21.2±21.3	5	7.5±9	4	0.2543
ΔInsulin, uU/ml	-0.8±4.9	22	0.05±2.9	28	0.7±4.7	7	-1.7±1.4	6	2.7±2.6	5	3.1±4.6	4	0.1176
ΔGLP-1, pg/ml	-3.3±34.5	22	-0.5±75.3	28	0.8±52	5	-1.3±72	6	70.6±100.4	5	5.1±31.4	4	0.251
ΔLeptin, ng/ml	-4.8±14	22	-2.2±10.7	28	-3±5.9	7	1.1±19.2	6	-0.6±12.1	5	4.9±13.1	4	0.6776
ΔNEFAs, mEq/l	0.1±0.2 (34.2)	22	0.1±0.2 (35.4)	28	0.04±0.2 (26.3)	6	0.3±0.1 (56.8)	6	0.2±0.2 (46.8)	5	-0.1±0.2 (20)	4	**0.0441**
ΔAdiponectin, ug/ml	0.1±3.6	22	0.5±4.9	28	-0.9±5.1	6	7±8.4	6	0±2.6	4	2.8±4.3	4	0.2278
ΔQUICKI	-0.01±0.1	22	-0.01±0.1	26	0.04±0.1	7	0.02±0.03	6	-0.03±0.02	5	-0.01±0	4	0.4459
ΔHOMA-β	-35.8±62.3	22	-7.7±49.3	27	-27.2±22.2	7	-52.1±55.6	6	-8.4±25.1	5	7±57.3	4	0.2961
ΔHOMA-IR	0±1.1	22	0.1±0.6	27	0.6±1.6	7	-0.2±0.3	6	1.1±0.9	5	0.9±1.1	4	0.0773
ΔHunger, mm	1.2±13.3	21	1.2±13	28	2.4±16.2	8	-6±15	6	-1.7±12.6	5	3.2±20.4	4	0.9147
ΔFullness, mm	-0.7±15.2	21	0.2±12.9	28	-5.4±16.3	8	-2.1±17.3	6	8.2±11.6	5	-8.6±6.9	4	0.4805
ΔDesire to eat, mm	1.3±12.5	21	3.7±13.1	28	1.9±13.9	8	-4.8±13	6	-1.1±12.2	5	5.1±21.1	4	0.6883
ΔProspective consumption, mm	-0.4±14.6	21	3.6±13.8	28	2.1±16.3	8	-0.4±12.5	6	-2.7±6.8	5	7.7±13.3	4	0.789

Values are means ± SDs of change (Δ, baseline-week 8) in outcomes. Positive values indicate decline over 8 weeks whereas negative values indicate increase over 8 weeks. Analysis was performed using a Kruskal-Wallis test with snack (almond or cracker) x BMI (normal weight, overweight or obese) as between subject factor. Pairwise comparisons were performed using Dunn joint ranks method. Values in bold are $P<0.05$. Different letters between snack x BMI categories within each variable indicate significant ($P<0.05$) difference. The mean rank scores obtained from the Kruskal-Wallis test for significant variables are presented in brackets. HOMA- homeostasis model assessment, IR-insulin resistance, QUICKI-quantitative insulin sensitivity check

Table S4: Anthropometric, clinical, cardiovascular and appetite outcome changes over 8 weeks (baseline-week 8) by snack group and baseline fasting total cholesterol category

	Total cholesterol < 170 mg/dl				Total cholesterol ≥ 170 mg/dl				P-values
	Cracker		Almond		Cracker		Almond		
	Mean±SD	N	Mean±SD	N	Mean±SD	N	Mean±SD	N	(Group x cholesterol)
ΔBody mass, kg	2.5±12	12	3.8±22	22	2.8±23	23	4.1±16	16	0.8036
ΔFat mass, kg	3±12	12	3.3±22	22	8.4±23	23	5.8±16	16	0.9052
ΔFat free mass, kg	3.1±12	12	2.6±22	22	3.6±23	23	4.7±16	16	0.938
ΔWaist circumference, cm	1±12	12	0.7±22	22	0.9±23	23	0.7±16	16	0.2345
ΔSystolic blood pressure, mmHg	6.3±12	12	7.7±22	22	14.1±23	23	15.4±16	16	0.1877
ΔDiastolic blood pressure, mmHg	6.1±12	12	6.9±22	22	6.2±23	23	8.8±16	16	0.3732

ΔTriglycerides, mg/dl	17.7±11	11	31.6±21	21	56.7±23	23	85.5±16	16	0.9655
ΔGlucose, mg/dl	11.3±11 (32.7)	11	10.4±21 (26.1)a	21	14.7±23 (46.8)b	23	9.7±16 (35.7)	16	**0.0096**
ΔInsulin, uU/ml	2.8±11	11	2.8±22	22	5.3±23	23	3.4±16	16	0.2834
ΔGLP-1, pg/ml	27.7±10	10	83.9±22	22	65.5±22	22	43.3±16	16	0.137
ΔLeptin, ng/ml	14.5±11	11	12.4±22	22	11.4±23	23	12.4±16	16	0.3323
ΔNEFAs, mEq/l	0.2±11	11	0.2±22	22	0.2±22	22	0.2±16	16	0.8335
ΔAdiponectin, ug/ml	3.7±11	11	5.6±22	22	3.6±21	21	6.1±16	16	0.0995
ΔQUICKI	0.1±11	11	0.1±20	20	0.1±23	23	0.1±16	16	0.1339
ΔHOMA-β	22.3±11	11	53.1±21	21	60.5±23	23	48.4±16	16	0.0527
ΔHOMA-IR	0.8±11	11	0.6±21	21	1.4±23	23	0.8±16	16	0.3311
ΔHunger, mm	9.3±11	11	14.3±22	22	15.1±23	23	14.1±16	16	0.8705
ΔFullness, mm	13±11	11	13.8±22	22	16.4±23	23	11±16	16	0.1766
ΔDesire to eat, mm	7.3±11	11	14.7±22	22	14.3±23	23	13.2±16	16	0.9193
ΔProspective consumption, mm	8.1±11	11	15.3±22	22	16.1±23	23	10.3±16	16	0.5718

Values are means ± SDs of change (Δ, baseline-week 8) in outcomes. Positive values indicate decline over 8 weeks whereas negative values indicate increase over 8 weeks. Analysis was performed using a Kruskal-Wallis test with snack (almond or cracker) x total cholesterol categories (< 170 mg/dl or ≥ 170 mg/dl) as between subject factor. Pairwise comparisons were performed using Dunn joint ranks method. Values in bold are P<0.05. Different letters between snack x cholesterol categories within each variable indicate significant (P<0.05) difference. The mean rank scores obtained from the Kruskal-Wallis test for significant variables are presented in brackets. HOMA- homeostasis model assessment, IR-insulin resistance, QUICKI- quantitative insulin sensitivity check

Table S5: Anthropometric, clinical, cardiovascular and appetite outcome changes over 8 weeks (baseline-week 8) by snack group and baseline fasting glucose category

	Glucose < 100 mg/dl				Glucose ≥ 100 mg/dl				P-values
	Cracker		Almond		Cracker		Almond		
	Mean±SD	N	Mean±SD	N	Mean±SD	N	Mean±SD	N	(Group x glucose)
ΔBody mass, kg	-1.2±2.4	13	-2.4±4.1	23	-1.6±2.9	22	-1.6±3.8	14	0.8977
ΔFat mass, kg	-3.2±10.2	13	-1.3±4.1	23	-0.3±3.9	22	0.7±4.9	14	0.481
ΔFat free mass, kg	-1±3.3	13	-1.1±3.1	23	-1.3±3.5	22	-2.3±4.3	14	0.7644
ΔWaist circumference, cm	-0.1±1.1	13	0.1±0.7	23	-0.1±0.9	22	0.2±0.8	14	0.6878
ΔSystolic blood pressure, mmHg	-2.9±16.9	13	4.6±14.1	23	0.7±8.3	22	3.7±7.1	14	0.6425

ΔDiastolic blood pressure, mmHg	0.7±6.5	13	-0.2±8.8	23	-1.7±5.8	22	-0.2±5.4	14	0.3728
ΔTotal cholesterol, mg/dl	25.3±13.7 (31.5)	13	15.6±17.1 (22.4)a	23	52.3±33.9 (49)b	21	42.9±32 (43.2)b	14	**0.0001**
ΔLDL cholesterol, mg/dl	15.5±11 (33.2)	13	9.4±12.3 (24.4)a	23	27.2±21.6 (45.4)b	21	23.8±21.6 (43.7)b	14	**0.0032**
ΔHDL cholesterol, mg/dl	11.5±8.6 (34.8)	13	4.8±8 (19.6)a	23	22.9±13.6 (49.8)b	21	18.3±12.7 (43.4)b	14	**<0.0001**
ΔTriglycerides, mg/dl	-8.6±22.8	13	6.5±31.3	23	11±56.8	21	4.3±91.2	14	0.1229
ΔInsulin, uU/ml	-0.8±2.5	13	0.5±2.6	23	0.5±5.6	21	-0.2±3.6	14	0.0967
ΔGLP-1, pg/ml	6.2±26.3	11	7.5±83.6	23	10.3±67.7	21	-12±43.8	14	0.5955
ΔLeptin, ng/ml	-2.4±5.7	13	-2±13.2	23	-4.7±15.1	21	1±11.6	14	0.2876
ΔNEFAs, mEq/l	0.1±0.2	12	0.2±0.2	23	0.1±0.2	21	0.1±0.2	14	0.8496
ΔAdiponectin, ug/ml	0.3±3.5	12	2.6±6.9	23	-0.3±3.9	20	0.2±3.8	14	0.6019
ΔHunger, mm	3.9±8.9	12	-1.5±14.1	23	-0.5±15.5	22	4.4±13.3	14	0.3549
ΔFullness, mm	3±16.5	12	1.7±13.5	23	-2.4±14.4	22	-6.6±11.1	14	0.0573
ΔDesire to eat, mm	3.3±7.9	12	1.4±14.5	23	-0.03±14.3	22	5.4±13	14	0.5841
ΔProspective consumption, mm	2.7±10	12	2.3±14.5	23	-1.7±15.7	22	6.4±11.1	14	0.2533

Values are means ± SDs of change (Δ, baseline-week 8) in outcomes. Positive values indicate decline over 8 weeks whereas negative values indicate increase over 8 weeks. Analysis was performed using a Kruskal-Wallis test with snack (almond or cracker) x glucose categories (<100 mg/dl or ≥ 100 mg/dl) as between subject factor. Pairwise comparisons were performed using Dunn joint ranks method. Values in bold are P<0.05. Different letters between snack x glucose categories within each variable indicate significant (P<0.05) difference. The mean rank scores obtained from the Kruskal-Wallis test for significant variables are presented in brackets.

Table S6: Non-responders and responders of specific outcomes in the cracker and almond groups.

	Cracker		Almond		P-values
	Non-responders	Responders	Non-responders	Responders	
Body mass	26 (74)	9 (26)	28 (74)	10 (26)	0.9533
Waist circumference	24 (69)	11 (31)	18 (47)	20 (53)	0.0657
Total cholesterol	1 (3)	33 (97)	4 (11)	33 (89)	0.3591*
LDL cholesterol	1 (3)	33 (97)	8 (22)	29 (78)	**0.029***
HDL cholesterol[a]	33 (97)	1 (3)	30 (81)	7 (19)	0.0569*
Triglycerides	12 (35)	22 (65)	14 (38)	23 (62)	0.8241

Glucose	5 (15)	29 (85)	9 (24)	28 (76)	0.3056
HOMA-IR	13 (38)	21 (62)	16 (43)	21 (57)	0.6679
Systolic blood pressure	14 (40)	21 (60)	13 (34)	25 (66)	0.6088
Diastolic blood pressure	17 (49)	18 (51)	20 (53)	18 (47)	0.7288

Values are n (%). Responders are individuals that demonstrated a decline in outcomes (except HDL cholesterol) over 8 weeks. Non-responders are individuals that had no change/increase in outcomes (except HDL cholesterol) over 8 weeks. a, responders for HDL are categorized as individuals that had an increase/no change in HDL over 8 weeks and non-responders are individuals that demonstrated a decline in HDL over 8 weeks. Values in bold are P<0.05. Analysis was performed using a Chi-squared test. *, Fisher's exact test.

Table S7: P-values for Figures 2, 3, 4, 5, S1 and S2 main effects.

	Main effects of intervention week analysis			
	Group	Week	Group x week	Figure no.
Total cholesterol, mg/dl	0.9648	**<.0001**	0.0892	2
LDL cholesterol, mg/dl	0.6036	**<.0001**	0.2369	2
HDL cholesterol, mg/dl	0.333	**<.0001**	**0.0131**	2
Triglycerides, mg/dl	0.8578	**0.0073**	0.6937	2
Glucose, mg/dl	0.0271 (NS adj.)	**<.0001**	0.0264	3
Insulin[ƚ], uU/ml	0.0668	**0.0119**	0.3652	3
GLP-1[ƚ], pg/ml	0.807	**<.0001**	0.1385	3
Leptin[ƚ], ng/ml	0.4745	0.2127	0.5048	S1
NEFAs, mEq/l	0.9844	**<.0001**	0.549	S1
Adiponectin[ƚ], ug/ml	0.0464 (NS adj.)	**0.0011**	0.175	S1
	Main effects of GTT time points analysis			
	Group	Time	Group x time	
Glucose[ƚ], mg/dl	0.0192	**<.0001**	0.2119	4
Insulin[ƚ], uU/ml	0.0701	**<.0001**	0.0662	4
C-peptide[ƚ], ug/l	0.1113	**<.0001**	**0.0091**	**4**
GLP-1[ƚ], pg/ml	0.9973	**<.0001**	0.5926	4
Adiponectin[ƚ], ug/ml	0.0366 (NS adj.)	**<.0001**	0.4485	S2
Glucagon, pg/ml	0.4347	0.9218	0.1877	S2
NEFAs[ƚ], mEq/l	0.6962	**<.0001**	0.4991	S2
	Group effect of AUC and indices analysis			

Glucose, mg/dl x 120 min	**0.0223**	4
Insulin, uU/ml x 120 min	**0.0411**	4
C-peptide, ug/l x 120 min	**0.0337**	4
GLP-1, pg/ml x 120 min	0.8181	4
Adiponectin, ug/ml x 120 min	**0.1075**	S2
NEFAs, mEq/l x 120 min	0.4249	S2
Glucagon, pg/ml x 120 min	0.3855	S2
IRI	**0.0193**	5
Matsuda index	**0.0239**	5
Disposition index	0.9031	5

Values are P-values. Values in bold are P<0.05. NS adj.: non-significant after baseline/time 0 adjustment. AUC: area under the curve, GTT: glucose tolerance test, IRI-insulin resistance index. ł, variables transformed prior to analysis using Johnson's family of transformations

Table S8: OGTT area under the curves and indices by snack group and baseline BMI category

	Normal weight				Overweight				Obese				P-values
	Cracker		Almond		Cracker		Almond		Cracker		Almond		
	Mean±SD	N	Mean±SD	N	Mean±SD	N	Mean±SD	N	Mean±SD	N	Mean±SD	N	(Group x BMI)
Adiponectin, ug/ml x 120 min	1497.3±455.2	11	1925.1±471.1	13	1478.3±245.6	4	1465.9±558.2	5	1313.6±707.3	5	1034.7±497	2	0.1113
C-peptide, ug/l x 120 min	235223.8±61293.4	11	181393.4±88985.6	13	225224.3±38345.1	4	238518.4±70128.3	5	278756±98636.8	5	224278.5±122322	2	0.1212
GLP-1, pg/ml x 120 min	6630.7±1479.9	11	6501.5±1795.7	13	6444.2±1356	4	5636.6±1127.5	5	6254.7±1827.8	5	8966.2±3586.4	2	0.6949
Glucagon, pg/ml x 120 min	755.4±218	9	653.3±551.2	10	642.8±317	4	668.1±0	1	657.8±654.9	5	391±0	1	0.4224
Glucose, mg/dl x 120 min	15970.2±2313.2 (24.5)	11	13168.8±2093.7 (12.2)	13	18819.4±4761.3 (30.3)	4	15169.5±2321.6 (21.6)	5	15135±2531.1 (21)	5	18240±4974.5 (29)	2	**0.0398**
Insulin, uU/ml x 120 min	13219.9±3578	11	7736.3±5721.1	13	11480.9±5359.5	4	13664.2±5204.4	5	15575.2±6313.4	5	13050.4±7728.6	2	0.0641

NEFAs, mEq/l x 120 min	23.9±5.5	11	23±8.3	13	18.8±3.7	4	21.1±3.7	5	27.8±8.1	5	33.6±25.9	2	0.5113
IRI	212912541.7±67730869.6 (24.8)	11	105235746.2±78990967.1 (11.5)	13	227219229.3±156320762.3 (23)	4	215765213.2±107874543.2 (24.8)	5	247456160.4±132799644.7 (26.4)	5	257261852.5±205888653.4 (24.5)	2	**0.0422**
Matsuda index	4.7±2.7 (18.3)	11	10.2±4.9 (31)a	13	4.5±2.9 (17)	4	3.4±1.1 (13.8)	5	3.1±1.3 (11.4)b	5	2.7±2.1 (11)	2	**0.0041**
Disposition index	11.5±35.9	11	2.8±44.2	13	-11.8±42.8	4	16.3±12.5	5	7.5±15.5	5	6±6.7	2	0.7254

Values are means ± SDs. Analysis was performed using a Kruskal-Wallis test with snack (almond or cracker) x BMI (normal weight, overweight or obese) as between subject factor. Pairwise comparisons were performed using Dunn joint ranks method. Values in bold are P<0.05. Different letters between snack x BMI categories within each variable indicate significant (P<0.05) difference. The mean rank scores obtained from the Kruskal-Wallis test for significant variables are presented in brackets. IRI-insulin resistance index.

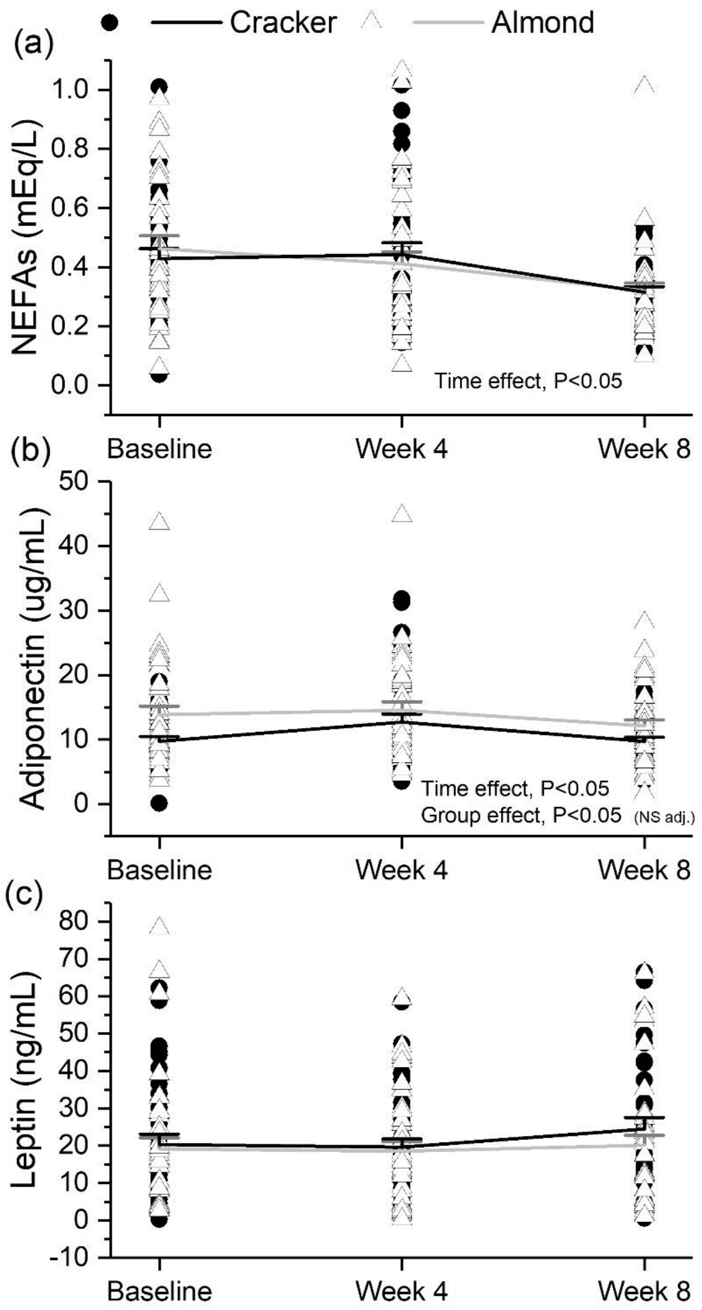

Cracker
Almond
(a)
NEFAs (mEq/L)
1.0
0.8
0.6
0.4
0.2
0.0
Time effect, P<0.05
Baseline
Week 4
Week 8
(b)
Adiponectin (ug/mL)
50
40
30
20
10
0
Time effect, P<0.05
Group effect, P<0.05
(NS adj.)
Baseline
Week 4
Week 8
(c)
Leptin (ng/mL)
80
70
60
50
40
30
20
10
0
-10
Baseline
Week 4
Week 8

Figure S1: Fasting (a) serum non-esterified fatty acids (NEFAs), (b) serum adiponectin and (c) plasma leptin profiles of the cracker and almond groups at baseline, week 4 and week 8 of the intervention.

Values are individual data points representing each participant at baseline, week 4 and week 8. Means ± SDs of the 2 snack groups at baseline, week 4 and week 8 are also plotted. Analysis was conducted using a linear mixed effects model with time as within-subject factor and snack group as between subject factor. Leptin and adiponectin values were transformed prior to analysis using Johnson's family of transformations. Cracker: N=35, Almond: N=38. Adiponectin time effect: baseline vs. week 4, $P<0.05$; week 4 vs. week 8, $P<0.05$. NEFAs time effect: baseline vs. week 8, $P<0.05$; week 4 vs. week 8, $P<0.05$. NS adj.: non-significant after baseline adjustment.

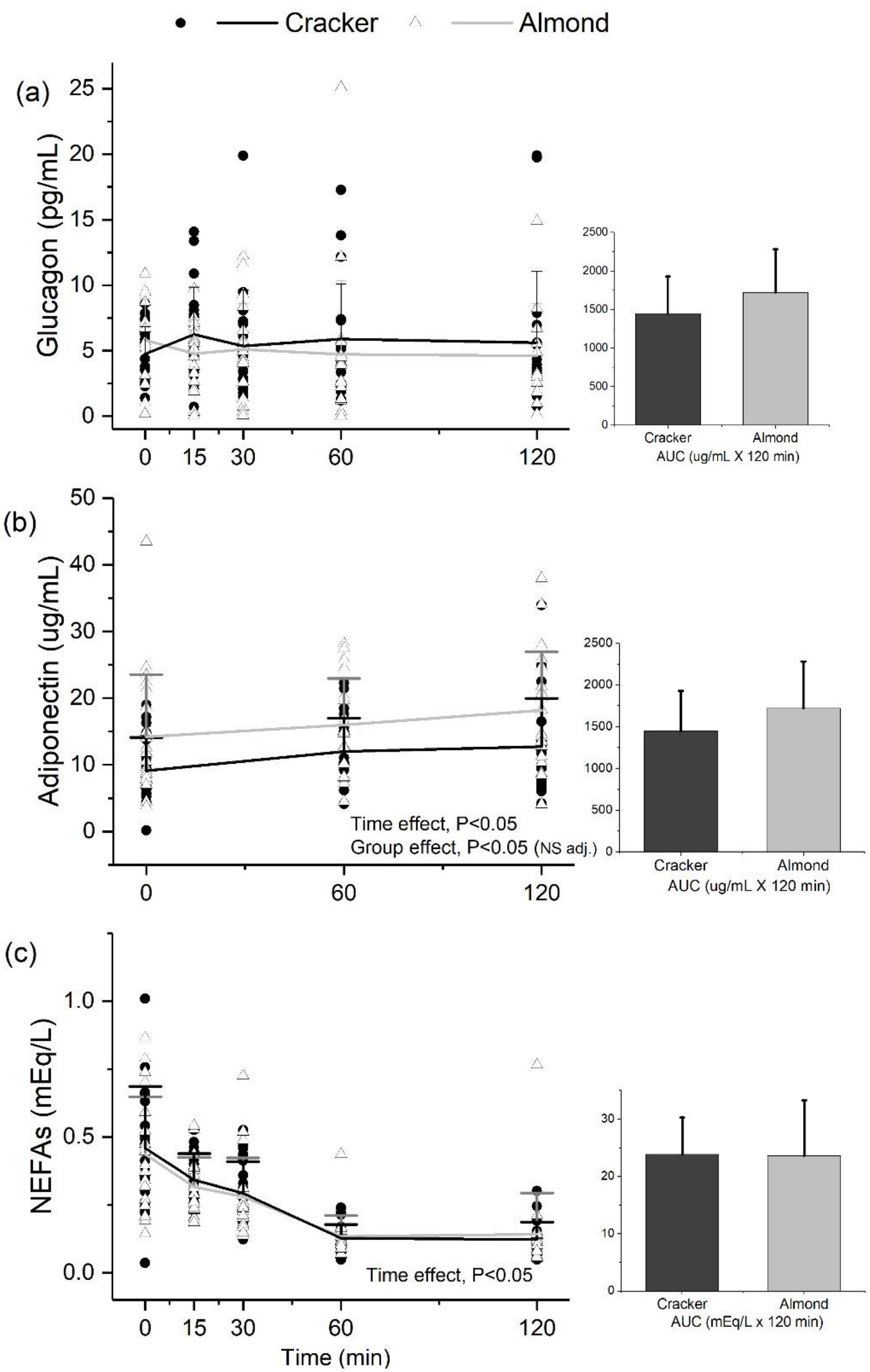

Cracker
Almond
(a)
Glucagon (pg/mL)
AUC (ug/mL X 120 min)
(b)
Adiponectin (ug/mL)
Time effect, P<0.05
Group effect, P<0.05 (NS adj.)
AUC (ug/mL X 120 min)
(c)
NEFAs (mEq/L)
Time effect, P<0.05
Time (min)
AUC (mEq/L x 120 min)

Figure S2: Serum (a) glucagon, (b) adiponectin, (c) and non-esterified fatty acids (NEFAs) profiles at 0, 15, 30, 60 and 120 minutes of an oral glucose tolerance test (OGTT) in the cracker and almond groups at week 8 of the intervention.

Values are individual data points representing each participant at each time point. Means ± SDs of the 2 snack groups at each time point are also plotted. Analysis was conducted using a linear mixed effects model with time as within-subject factor and snack group as between subject factor. Adiponectin and NEFAs values were transformed prior to analysis using Johnson's family of transformations. Area under the curves (Means ± SDs) are displayed adjacent to the respective OGTT profiles. Analysis was conducted using a one-way ANOVA with snack group as between subject factor. Cracker: N=20, Almond: N=20. Adiponectin time effect: 60, 120 vs. 0 min. NEFAs time effect: 30, 60, 120 vs. 0 min; 30, 60, 120 vs. 15 min; 60, 120 vs. 30 min, $P<0.05$. NS adj.: non-significant after time 0 adjustment.

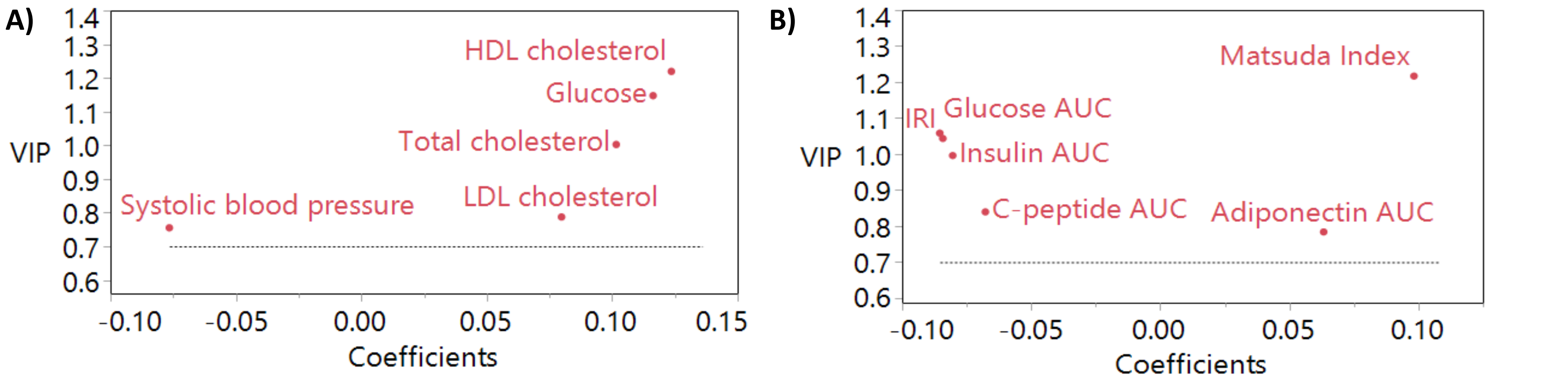

Figure S3: Variable importance projection (VIP) score vs coefficient plot from the pruned partial least squares discriminant analyses (PLS-DA) A) intervention snack model, B) OGTT snack model.

Partial least squares-discriminant analysis (PLS-DA) was conducted on: A) changes in anthropometric and clinical outcomes over 8 weeks, and B) OGTT AUCs and insulin sensitivity/resistance indices, to determine which features were the most discriminatory (VIP score > 0.7) between the snack groups. Input dataset for the intervention analysis comprised of changes in BM, fat mass, fat-free mass, waist circumference, systolic and diastolic blood pressure, total, HDL and LDL cholesterol, triglycerides, NEFAs, adiponectin, GLP-1, insulin, leptin, glucose, HOMA-IR, HOMA-β, QUICKI, and appetite ratings over the 8-week intervention. Input dataset for the OGTT analysis comprised of HOMA-IR, HOMA-β, QUICKI, IRI, DI, Matsuda index, and AUCs for adiponectin, GLP-1, insulin, C-peptide, NEFAs, glucose, and glucagon. Data were standardized (i.e. centered and scaled) prior to PLS-DA and the model was fit with SIMPLS algorithm. The model was pruned to determine best fit and validated with leave-one-out old cross validation. Principal component analysis (PCA) was conducted on the correlation matrix obtained from the discriminatory features (identified by PLS-DA) for visualization purposes.

AUC: area under the curve, HOMA-IR: homeostatic model assessment-insulin resistance, IRI; insulin resistance index; QUICKI: quantitative insulin-sensitivity check index, OGTT: oral glucose tolerance test

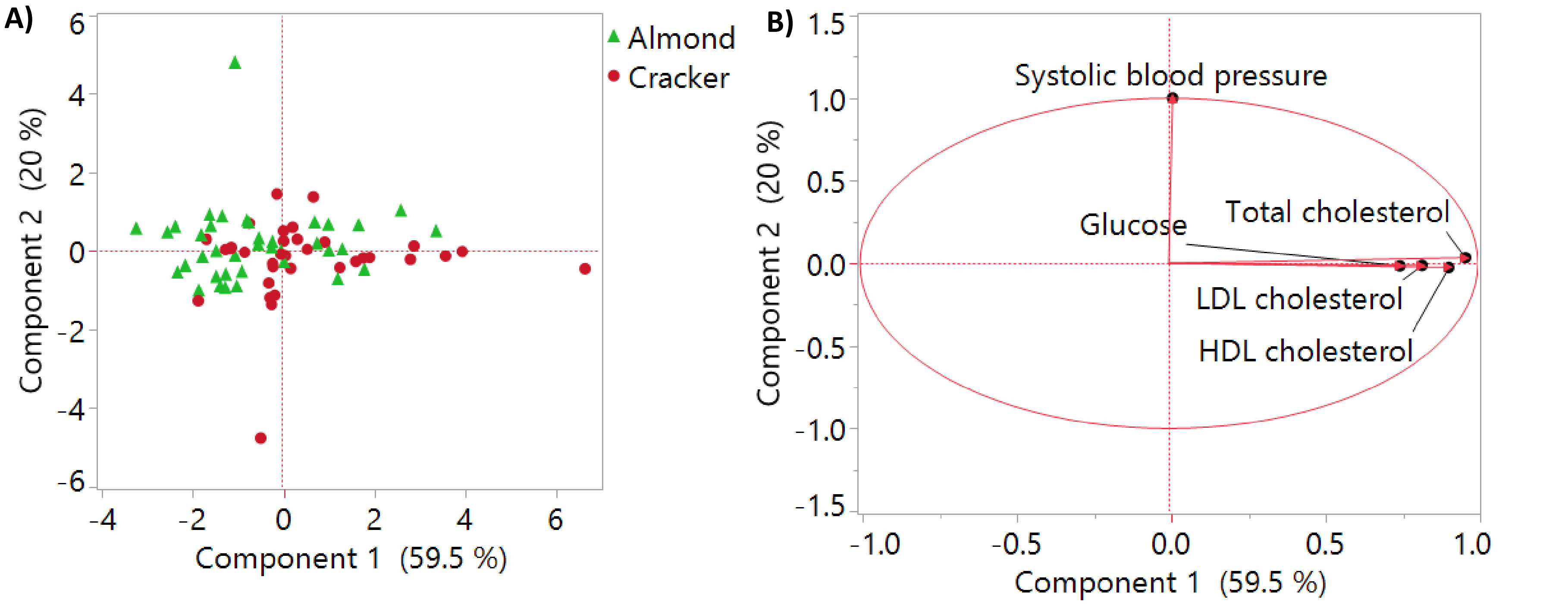

Figure S4: Principal component analysis A) score plot of participants and B) loading plot of variables- i.e. Δglucose, Δtotal cholesterol, ΔLDL cholesterol, ΔHDL cholesterol and Δsystolic blood pressure identified by PLS-DA as most discriminatory between the snack groups over the 8-week intervention. Δ, baseline-week 8.

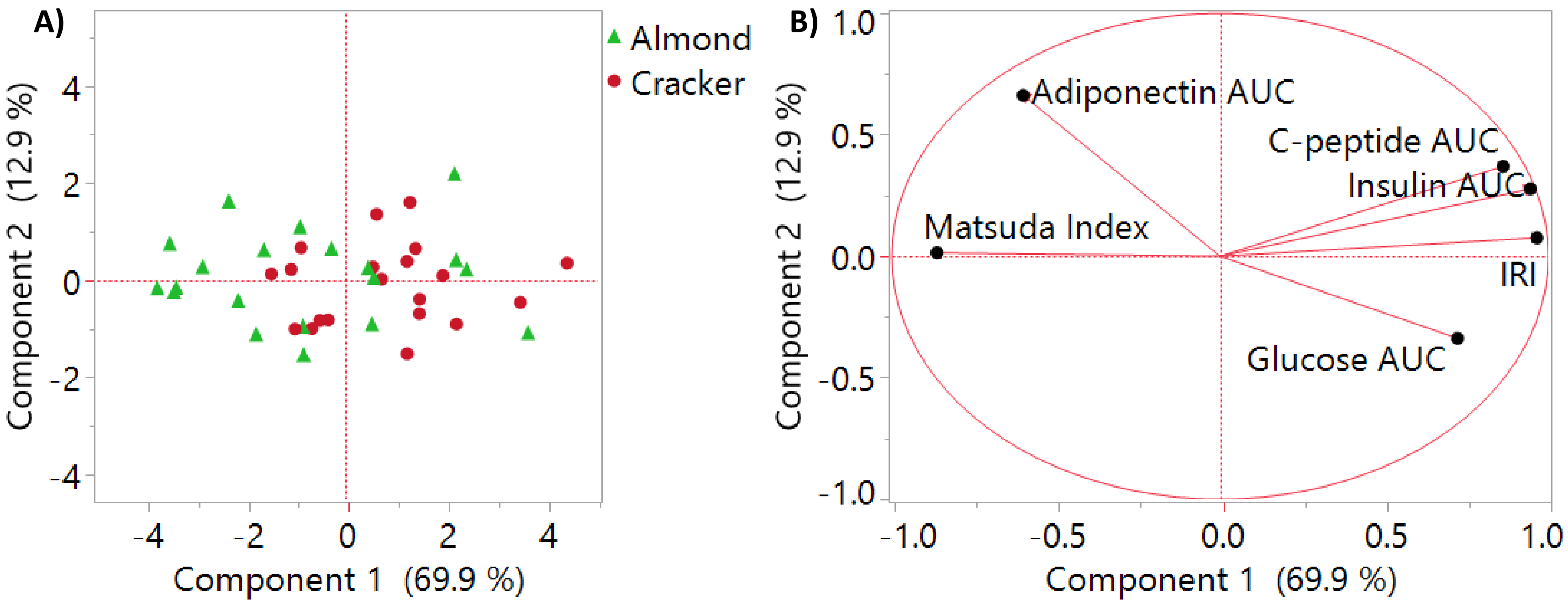

Figure S5: Principal component analysis A) score plot of participants and B) loading plot of variables i.e. adiponectin AUC, C-peptide AUC, insulin AUC, glucose AUC, IRI and Matsuda index identified by PLS-DA as most discriminatory between the snack groups during the OGTT.

AUC: area under the curve, IRI; insulin resistance index; OGTT: oral glucose tolerance test
